# Genetic diversity and linkage disequilibrium using SNP (KASP) and AFLP markers in a worldwide durum wheat (*Triticum turgidum* L. var *durum*) collection

**DOI:** 10.1371/journal.pone.0218562

**Published:** 2019-06-28

**Authors:** Pablo Federico Roncallo, Valeria Beaufort, Adelina Olga Larsen, Susanne Dreisigacker, Viviana Echenique

**Affiliations:** 1 Centro de Recursos Naturales Renovables de la Zona Semiárida (CERZOS–CCT–CONICET Bahía Blanca) and Departamento de Agronomía, Universidad Nacional del Sur, Bahía Blanca, Argentina; 2 CEI Barrow, Instituto Nacional de Tecnología Agropecuaria (INTA), Tres Arroyos, Buenos Aires, Argentina; 3 International Maize and Wheat Improvement Center (CIMMYT), El Batán, Edo. de México, México; Instituto Agricultura Sostenible, SPAIN

## Abstract

The aim of this work was to analyze the genetic diversity and linkage disequilibrium in a collection of 168 durum wheat accessions (*Triticum turgidum* L. var. *durum*) of different origins. Our collection was mainly composed of released and unreleased Argentinian germplasm, with additional genotypes from Italy, Chile, France, CIMMYT, Cyprus, USA and WANA region. To this end, the entire collection was characterized with 85 Single Nucleotide Polymorphism (SNP) markers obtained by Kompetitive Allele Specific PCR (KASP), giving a heterozygosity (*He*) mean value of 0.183 and a coefficient of genetic differentiation (*Gst*) value of 0.139. A subset of 119 accessions was characterized with six Amplified Fragment Length Polymorphism (AFLP) primer combinations. A total of 181 polymorphic markers (125 AFLP and 56 SNP) amplified across this subset revealed *He* measures of 0.352 and 0.182, respectively. Of these, 134 were selected to estimate the genome-wide linkage disequilibrium obtaining low significant values (*r*^*2*^ = 0.11) in the subset, indicating its suitability for future genome-wide association studies (GWAS). The structure analysis conducted in the entire collection with SNP detected two subpopulations. However, the structure analysis conducted with AFLP markers in the subset of 119 accessions proved to have greater degree of resolution and detect six subpopulations. The information provided by both marker types was complementary and showed a strong association between old Argentinian and Italian germplasm and a contribution of CIMMYT germplasm to modern Argentinian, Chilean and Cypriot accessions. The influence of Mediterranean germplasm, mainly from Italy, on part of the modern Argentinian cultivars or breeding lines was also clearly evidenced. Although our analysis yields conclusive results and useful information for association mapping studies, further analyses are needed to refine the number of subpopulations present in the germplasm collection analyzed.

## Introduction

Durum wheat (*Triticum turgidum* L. var *durum*) is the most important tetraploid wheat species and the raw material for pasta and semolina production. The durum wheat area in Argentina is located mainly in the southeast of Buenos Aires province where it grows under rain-fed conditions without supplementary irrigation. In the last four decades, this cultivated area has been reduced from 430,000 to an average of 65,466 hectares mainly as a result of the loss of competitiveness against common wheat (http://datosestimaciones.magyp.gob.ar/). Argentinian durum breeding programs are currently being conducted by public organisms, such as the Instituto Nacional de Tecnología Agropecuaria (INTA) and two private companies. However, the number of commercially released durum wheat cultivars during the last 20 years has nonetheless been low and only ten cultivars are normally available for farmers in the seed market. Seven new cultivars were released in 2017 and new breeding companies have got involved in durum wheat breeding.

Given the narrow base of durum wheat germplasm sown in the country, the maintenance or increase of genetic diversity within local durum wheat breeding programs is crucial to the successful improvement of the crop. Furthermore, the introgression of new variability into germplasm in local breeding programs not only can increase rates of genetic gain but also avoid outbreaks of either new diseases or pathogen races. However, in order to expand the genetic base used by local breeding programs, the existing genetic diversity has initially to be assessed before a proper conservation and utilization strategy can be defined and deployed. In addition, varietal identification and differentiation are also important to guarantee seed purity and classification during storage for industry. Both the breadth of the genetic base and the breeding strategy will finally determine breeding success.

Different methods are available for the assessment of genetic diversity among accessions. Traditional methods based on phenotypic characterization and pedigree analyses have proved not to be sufficiently accurate to estimate detailed genetic relationships among germplasm [1]. In addition, phenotypic traits are limited in number and they may be affected by environmental conditions [2]. For these reasons, genotyping has emerged as a convenient tool to assess genetic diversity in a germplasm collection. The adoption of marker technologies to characterize germplasm or its use in marker-assisted selection is still incipient in our national durum wheat breeding programs, while increasingly routine in private and public breeding programs globally. Previous reports have already demonstrated that different types of molecular markers are able to resolve genetic relationships between durum wheat accessions [1, 3–8]. However, estimated genetic relationships are not always comparable when using different marker technologies [9]. In parallel, the identification of new cultivars or breeding materials through molecular markers is a useful tool for the protection of breeders’ rights [10].

Simple Sequence Repeat (SSR) markers are multi-allelic and thus have the ability to capture higher variability than e.g. biallelic markers like Single Nucleotide Polymorphism (SNP) [11] or Amplified Fragment Length Polymorphism (AFLP) [12] and Diversity Arrays Technology (DArT) markers [13]. The AFLP technique, in particular, has the ability to produce a large number of polymorphic bands per single lane (genotype) with the additional advantage of lower initial costs and higher transferability across species with respect to the SSR marker technique [14]. Other authors suggested that the high number of polymorphic loci detected by AFLP could counterbalance the loss of information resulting from their dominant nature [15]. More recently, a shift towards the use of SNP markers instead of microsatellites (SSRs) has occurred [16]. Even though, some authors [17] suggest that AFLP and SSR markers are more suitable for diversity analysis and fingerprinting. Furthermore, SNP markers are the most abundant polymorphisms in any species [18, 19]. Therefore, its identification and use are frequent particularly in those crops for which the entire genome sequence is available [20].

The development of multiplexed array-based high-throughput genotyping technologies [21] or uniplex SNP genotyping platforms, such as TaqMan^TM^ [22] and KASP^TM^, among others, has greatly facilitated the use of SNP markers. Kompetitive Allele Specific PCR or KASP^TM^ is a cost-effective technology with high sensitivity, accuracy and flexibility allowing genotyping either few samples with many SNP markers or several samples with few SNP markers in a single plate [11]. This technology is suitable in applications that require a low to moderate number of markers. As stated above, and due to their intrinsic properties, each marker type can provide different levels of information.

On the other hand, the study of a germplasm collection can be addressed considering not only relatedness among genotypes but also analyzing the association among different loci across genotypes. This association can be measured through the pairwise correlation of allele frequencies in different loci. Linkage disequilibrium (LD) is the nonrandom association of alleles at two or more loci in a population [23]. The LD pattern is affected by most of the population genetics processes and is directly related to the mutation and recombination history of a population [24, 25]. The presence of related subgroups in the population referred to as population structure is a consequence of non-random mating among individuals resulting in an increase of LD, some individuals being more closely related than others [26, 27]. LD mapping or association mapping is a powerful tool based on LD to identify marker-trait associations (MTA) [28]. Knowledge on LD as well as on population structure is therefore an important requisite to be taken into account at the moment of designing and carrying out association mapping studies [29].

No previous studies implementing molecular markers have been carried out to date to estimate genetic diversity and linkage disequilibrium using the germplasm available in Argentinian durum wheat breeding programs. In this study, we characterized released cultivars, breeding lines and landraces from national breeding programs and foreign germplasm from different geographic regions using two molecular markers technologies. The aim of our work was to assess the genetic diversity and linkage disequilibrium present in our durum wheat collection using AFLP and SNP markers.

## Materials and methods

### Plant material and field trial

Plant material conformed by 168 accessions of diverse origins, included 62 genotypes from Argentina, 31 from Italy, 25 from Chile, 20 from France, 14 from West Asia and North Africa (WANA) region (Syria, Lebanon, Israel, Algeria, Libya, Sudan, Ethiopia), nine from CIMMYT, four from USA and three from Cyprus (Table 1). Most of the durum wheat accessions classified as old, modern cultivars and breeding lines were provided by the public breeding programs of INTA, Argentina; the Instituto de Investigaciones Agropecuarias-Quillamapu (INIA), Chile; the Agricultural Research Institute (ARI), Cyprus; and Argentinian private companies (ACA Semillas, Buck Semillas). Landraces (Etit 38, Haurani and Taganrog and additional old cultivars were provided by INTA´s public seed bank located in Marcos Juárez, Córdoba province, Argentina. With the exception of the accession DGE-1 and Langdon (DIC-3A) all accessions in the collection belong to *Triticum turgidum* L.var *durum* (2n = 4x = 28; AABB genome). DGE-1 (2n = 28 + 2) is an alien disomic addition line that possesses an additional pair of chromosomes from diploid wheatgrass, *Lophopyrum elongatum* (Host) Á., added to confer resistance to *Fusarium* [30]. Langdon (DIC-3A)-10, another accession, is a recombinant inbred chromosome line (RICL) of the cultivar Langdon crossed with Langdon (DIC-3A) [31]. Langdon (DIC-3A) is a derived line carrying a chromosome 3A substitution from wild emmer (*T*. *turgidum* L. var. *dicoccoides*) [32].

**Table 1:** List of the 168 durum wheat accessions (cultivars, breeding lines and landraces) analyzed in this study.

ID	Accession name	Origin^a^	Year of registration^b^	Pedigree	Donor^c^
1	Bonaerense Quilaco ^●^	ARM	1987	MAGHREBI-72/GANSO//ANHINGA/3/RABICORNO//D-21563/ANHINGA	INTA Marcos Juárez
2	Buck Cristal ^●^	ARM	1988	GAVIOTA/USA-01992[1765];SHASTA/USA-01992[1281]; (GTA/USA)	Buck Semillas
3	Buck Ambar ^●^	ARM	1995	TROB/4/FG/CIT//BBAL/3/CDK/CDEN//BBAL	Buck Semillas
4	BonINTA Cumenay ^●^	ARM	1995	CPP//TGBB/GDO 516	INTA Barrow
5	Buck Topacio ^●^	ARM	1997	PROB611/ALTAR 84	Buck Semillas
6	BonINTA Facon ^●^	ARM	1997	STN"S"/3/CHUR"S"/HUI"S"//POC"S"/4/MO"S"	INTA Barrow
7	Buck Esmeralda ^●^	ARM	2000	CDEU / BONQUI	Buck Semillas
8	Buck Platino ^●^	ARM	2002	BAMB"S"//MO"S"/YAV79	Buck Semillas
9	BonINTA Carilo ^●^	ARM	2002	TGBB/CANDEF/3/BERK/GDO VZ516//MTTE"S"/4/LAKOTA/CANDO	INTA Barrow
10	ACA 1801F ^●^	ARM	2008	BONQUILACO/BCANDISUR	A.C.A.
11	ACA 1901 F	ARM	2009	KOFA/UCD1113-LINE_199	A.C.A.
12	Buck Granate	ARM	2010	BCRIS//BBAL/BAMB"S"	Buck Semillas
13	BonINTA Quillen	ARM	2015	BICAR#9634/BONVAL	INTA Barrow
14	Buck Zafiro	ARM	2015	BTOP/4/CMH79.1159/YAV"S"/3/BBAL//CAPRI/BTOP	Buck Semillas
15	VF 0154 ^●^	ARM	nr	SORD 1/PLATA 16	INTA Bordenave
16	VF 042 ^●^	ARM	nr	SCAR"S"/DGOVZ579//CP/3/T.TURANICUM/BIN//GRANDUR	INTA Bordenave
17	VF 0113 ^●^	ARM	nr	LLOYD (USA 1983, CANDO/EDMORE)	INTA Bordenave
18	VF 0163 ^●^	ARM	nr	BI.FACON/BELFUGUITO	INTA Bordenave
19	VF 003 ^●^	ARM	nr	GANS"S"	INTA Bordenave
20	VF 0121 ^●^	ARM	nr	MTVD 10–98 HUNGRIA	INTA Bordenave
21	VF 0167 ^●^	ARM	nr	CDK/2620.89/PROB611/ALTAR 84	INTA Bordenave
22	VF 0136 ^●^	ARM	nr	CHEN/ALTAR 84/4/SRN//HUI/YAV79/3/SKARV/…	INTA Bordenave
23	VF 0137 ^●^	ARM	nr	PLATA10/6/MQUE/4/USDA573/QFN/AA-7/3/ALBA- D/5/AVO/HUI/7/PLATA_13/8/THKNEE_11/9/CHEN/ALTAR 84/3/HUI/POC//BUB/RUFO/4/FNFOOT	INTA Bordenave
24	B#24 ^●^	ARM	nr	TATLER-1/BEJAH-7	Buck Semillas
25	B#25 ^●^	ARM	nr	GDOVZ394//SBA81/PLC”S”/7/YEL”S”/BAR”S”/3/GR”S”/AFN//CR”S”/5/DON”S”//CR”S”*2/GS”S”/3/… (VEROLI)	Buck Semillas
26	B#27 ^●^	ARM	nr	BCRIS//BBAL/BAMB"S"	Buck Semillas
27	CBW 0105 ^●^	ARM	nr	BELFUGITTO//CATA"S"/STN"S"/3/LAKOTA/CANDO	INTA Barrow
28	CBW 0112 ^●^	ARM	nr	BELFUGITTO//CATA"S"/STN"S"/3/F.LUNGA/GDO 645	INTA Barrow
29	CBW 0120	ARM	nr	TOPAZ/CSLP/6/BR 180/3/ DK 60.120/LDS//64.210/4/BERK 469/5/ALTAR84/AOS "S"	INTA Barrow
30	CBW 0141 ^●^	ARM	nr	BONVAL//F.LUNGA/GDO 645	INTA Barrow
31	CBW 0153 ^●^	ARM	nr	BONVAL/BAMB	INTA Barrow
32	CBW 0200 ^●^	ARM	nr	BONVAL//F.LUNGA//GDO645/3/PROB611/ALTAR84	INTA Barrow
33	CBW 0210	ARM	nr	BONVAL/BAMB/3/SILVER_23/ARLIN_3//DON87	INTA Barrow
34	CBW 0225 ^●^	ARM	nr	BONQUI/BAMB/BIFAC	INTA Barrow
35	CBW 0230 ^●^	ARM	nr	CSLP/4/KURZSTROH//LEEDS/BIDI17/3/MONDUR/5/PROB611/ALTAR84	INTA Barrow
36	CBW 0001 ^●^	ARM	nr	INTER_18	INTA Barrow
37	CBW 0002 ^●^	ARM	nr	KNAR_3/MOJO_2//ACO89	INTA Barrow
38	CBW 0004 ^●^	ARM	nr	AVTA/YAZI_1	INTA Barrow
39	CBW 0101 ^●^	ARM	nr	BELFUGITTO//CATA”S”/STN”S”/3/LAKOTA/CANDO	INTA Barrow
40	CBW 0111 ^●^	ARM	nr	BELFUGITTO//CATA”S”/STN”S”/3/F.LUNGA/GDO 645	INTA Barrow
41	CBW 0156 ^●^	ARM	nr	BONVAL/BAMB	INTA Barrow
42	B33.1123.16-3-4-3	ARM	nr	BICRL/4/BONQUI/3/ALTAR84/FUUT"S"//AAZ"S"	Buck Semillas
43	DD26	ARM	nr	UC1113/KOFA	A.C.A.
44	DD150	ARM	nr	UC1113/KOFA	A.C.A.
45	CBW 05082	ARM	nr	BICAR#9634/BONVAL	INTA Barrow
46	CBW 05024	ARM	nr	BCRIS/BICUM"S"//BICAR#9639	INTA Barrow
47	CBW 05072	ARM	nr	BR180/3/DK60.120/LDS//64210/4/BERK/5/STIL"S"/YAV"S"/6/TGSB/GDO598/7/BICAR#9641	INTA Barrow
48	CBW 05081	ARM	nr	BICAR#9634/BONVAL	INTA Barrow
49	CBW 08131	ARM	nr	BCRIS/BICUM"S"//BICAR#9639/3/POHO_1//CETA/SRN_3	INTA Barrow
50	CBW 09034	ARM	nr	BONVAL/3/POHO_1//CETA/SRN_3	INTA Barrow
51	ACA 2125.07	ARM	nr	CBW40/KOFA	A.C.A.
52	ACA 4420.08	ARM	nr	ACA1801F/KOFA-10	A.C.A.
53	Taganrog ^●^	ART	1934	SOUTH RUSSIAN LANDRACE	INTA Marcos Juárez
54	Candeal Durumbuck ^●^	ART	1952	CANDEAL/TAGANROG	INTA Marcos Juárez
55	Taganrog Sel. BUCK ^●^	ART	1961	SELECCIÓN(20–42) DE TAGANROG COMÚN	INTA Marcos Juárez
56	Taganrog Vilela Fideos ^●^	ART	1961	TAGANROG NO.7 SELECTION	INTA Marcos Juárez
57	Balcarceno INTA ^●^	ART	1974	BBAL//BYE*2/TC60	INTA Marcos Juárez
58	Buck Mechongue ^●^	ART	1979	DT216.156//MOGH/WELLS/3/RL3442/LK/4/TACE/3*TC	INTA Marcos Juárez
59	Bonaerense Valverde ^●^	ART	1980	GIORGIO370//CAPELLI/YUMA (Gerardo 516)	INTA Marcos Juárez
60	Taganrog Buck Balcarce ^●^	ART	1980	CAPELLIX(CANDEAL ITALIANOX(CAND.XTAG. 17-13-4)	INTA Marcos Juárez
61	BF 1776 ^●^	ART	nr	GIORGIO//CAPELLI/YUMA	INTA Marcos Juárez
62	Buck No6 ^●^	ART	nr	YAV"S"/SCO"S"//STIL"S"	INTA Marcos Juárez
63	Llareta INIA	CHI	1997	D67.54.4.9A//JORI’S’/ROSNER DURUM 119-200-4Y/3/SAHEL77	INIA Chile
64	Corcolen INIA	CHI	2005	ALGA’S’/3/CANDEALFEN5/FLAMINGO’S’//PETREL’S’/4/CHURRILLAS’S’/5/AUK’S’/6/RUFF’S’/FLAMINGO’S’//FLAMINGO’S’/CRANE’S’/3/YAV79/HUITLES’S’	INIA Chile
65	Lleuque INIA	CHI	2011	YEL’S’/BAR’S’/3/GR’S’/AFN//CR’S’/5/DOM’S’// CR’S’*2/GS’S’/3/SCO’S’/4/HORA/6/LAP76/GULL’S’/7/LICAN	INIA Chile
66	Quc 3585–2007	CHI	nr	POHO1/4/ALTAR84/CMH84/CMH82A.1062//RISSA’S’/3/ACONCHI89	INIA Chile
67	Quc 3739–2008	CHI	nr	OSU-3880005/3/STOT//ALTAR84/ALD/4/KUCUK2/5/CRAKE10/RISSA	INIA Chile
68	Quc 3104–2005	CHI	nr	ALTAR84/ALD’S’//STN’S’/CHEN’S’/ALTAR84/4/ATES1D	INIA Chile
69	Quc 3587–2007	CHI	nr	POHO1/4/ALTAR84/CMH84/CMH82A.1062//RISSA’S’/3/ACONCHI89	INIA Chile
70	Quc 3693–2008	CHI	nr	GUAYACAN INTA//YUAN1/GREEN18/3/SOOTY9/RASCON 37	INIA Chile
71	Quc 3584–2007	CHI	nr	POHO1/4/ALTAR84/CMH84/CMH82A.1062//RISSA’S’/3/ACONCHI89	INIA Chile
72	Quc 3738–2008	CHI	nr	OSU-3880005/3/STOT//ALTAR84/ALD/4/KUCUK2/5/RASCON 37/2*TARRO2	INIA Chile
73	Quc 3506–2007	CHI	nr	ALTAR84/STINT’S’//SILVER/4/ALTAR84/CMH82A.1062//RISSA’S’/3/ACONCHI’S	INIA Chile
74	Quc 3755–2008	CHI	nr	VANRRIKSE6.2//1^**a**^-1D 2+12-5/3*WB881	INIA Chile
75	Quc 3672–2008	CHI	nr	SNITAN/3/STOT//ALTAR84/ALD	INIA Chile
76	Quc 3555–2007	CHI	nr	NACH’S’/CHEN’S’//RUFO’S’/ALD’S’/3/SQLA’S’/4/CRANE’S’/PLAC1485	INIA Chile
77	Quc 3694–2008	CHI	nr	GUANAY/3/STOT//ALTAR84/ALD/4/BINTEPE85/SULA	INIA Chile
78	Quc 3497–2007	CHI	nr	NACH’S’/CHEN’S’//RUFO’S’/ALD’S’/3/SQLA’S’/7/YEL’S’/BAR’S’/3/GR’S’/AFN// CR’S’/5/DOM’S’//CR’S’*2/GS’S’/3/SCO’S’/4/HORA/6/LAP76/GUIL’S’	INIA Chile
79	Quc 3509–2007	CHI	nr	ATES 2-D/7/YEL’S’/BAR’S’/3/GR’S’/AFN//CR’S’/5/DOM’S’//CR’S’*2/GS’S’ /3/SCO’S’/4/HORA /6/LAP76/GUIL’S’	INIA Chile
80	Quc 3538–2009	CHI	nr	na	INIA Chile
81	Quc 3730–2008	CHI	nr	na	INIA Chile
82	Quc 3775–2008	CHI	nr	ATES 1-D/LLARETA INIA	INIA Chile
83	Quc 3559–2009	CHI	nr	na	INIA Chile
84	Quc 3506–2009	CHI	nr	na	INIA Chile
85	Quc 3427–2009	CHI	nr	na	INIA Chile
86	Quc 3462–2009	CHI	nr	na	INIA Chile
87	Quc 3763–2008	CHI	nr	na	INIA Chile
88	Gallareta = Alta 84 ^●^	CIM	1982	RUFF/FLAMINGO-DW//MEXICALI-75/3/SHEARWATER/4/?	INTA Marcos Juárez
89	Gan ^●^	CIM	1983	GGOVZ355/GS//MEXI75	INTA Marcos Juárez
90	Focha ^●^	CIM	1991	SULA//WELLS/DWL5023	INTA Marcos Juárez
91	65-IAT2 ^●^	CIM	nr	AJAIA_12/F3LOCAL(SEL.ETHIO.135.85)//PLATA_13	A.C.A.
92	66-IAT2 ^●^	CIM	nr	CADO/BOOMER_33	A.C.A.
93	69-IAT2 ^●^	CIM	nr	PLATA_1/SNM//PLATA_9	A.C.A.
94	71-IAT2 ^●^	CIM	nr	SOOTY_9/RASCON_37	A.C.A.
95	73-IAT2 ^●^	CIM	nr	TOTUS/CARGO//ALTAR84/AOS	A.C.A.
96	80-IAT2 ^●^	CIM	nr	YAVAROS TALL	A.C.A.
97	Hekave	CYP	2003	DRA'S'//LLOYD/KIA	Cyprus A.R.I.
98	Ourania	CYP	2007	CULT.DW/T.DIC	Cyprus A.R.I.
99	Josephina	CYP	2007	LLOYD/KIA*3	Cyprus A.R.I.
100	Ardente ^●^	FRA	1984	ISRAEL DURUM 303/PRELIMINARY77//664	INTA Barrow
101	Neodur	FRA	1987	184-7/VALDUR//EDMORE	Buck Semillas
102	Alcalou ^●^	FRA	1990	VALSACCO/RANGER	INTA Barrow
103	Ixos	FRA	1990	VALNOVA/3/TOMCLEAR/662//662	INTA Barrow
104	Exeldur ^●^	FRA	1992	VALDUR/REGAL	Buck Semillas
105	Arbois ^●^	FRA	1996	na	INTA Barrow
106	Argeles ^●^	FRA	1996	na	Buck Semillas
107	Sachem ^●^	FRA	1999	na	Buck Semillas
108	Biensur ^●^	FRA	2000	na	Buck Semillas
109	Joyau ^●^	FRA	2001	na	Buck Semillas
110	Karur ^●^	FRA	2002	na	Buck Semillas
111	Durobonus ^●^	FRA	2004	na	Buck Semillas
112	Vivadur ^●^	FRA	2003	na	Buck Semillas
113	Arcodur	FRA	na	Na	INTA Barrow
114	Orlu ^●^	FRA	2001	na	Buck Semillas
115	Garic ^●^	FRA	na	na	Buck Semillas
116	Byblos ^●^	FRA	2003	na	Buck Semillas
117	Nautilur ^●^	FRA	na	na	Buck Semillas
118	Artimon ^●^	FRA	na	na	Buck Semillas
119	Amarillo	FRA	nr	na	Buck Semillas
120	Simeto ^●^	ITM	1988	CAPEITI 8/VALNOVA	A.C.A.
121	Italo ^●^	ITM	1993	COMPLEX CROSS BETWEEN ITALIAN AND TURKISH GENOTYPES TURCHIA//CRESO/CAPEITI-8	INTA Barrow
122	Colosseo ^●^	ITM	1995	MUTANTE DI MEXA/CRESO	A.C.A.
123	Fortore ^●^	ITM	1995	CAPEITI 8/VALFORTE	INTA Barrow
124	Ciccio ^●^	ITM	1996	F6 APPULO/VALNOVA//VALFORTE/PATRIZIO	A.C.A.
125	Cannizzo ^●^	ITM	1998	CAPEITI/VALNOVA/2/PATRICIO/VALFORTE	INTA Barrow
126	Concadoro ^●^	ITM	1998	SIMETO/2/CAPEITI/VALFORTE	INTA Barrow
127	Dupri ^●^	ITM	1998	DUILIO/PRIMADUR	Buck Semillas
128	Portorico ^●^	ITM	2000	AMBRAL X DUILIO	Buck Semillas
129	Tiziannia ^●^	ITM	2001	PELEO/NEODUR	Buck Semillas
130	Duetto ^●^	ITM	2002	1485/83-74	Buck Semillas
131	Catervo ^●^	ITM	2004	COLOSSEO/PLATANI	INTA Barrow
132	Core ^●^	ITM	2008	GIANNI/PLATANI	INTA Barrow
133	Cantico ^●^	ITM	na	PLATANI/GIANNI	INTA Barrow
134	Ci 1936 ^●^	ITM	nr	CICCIO/LÍNEA PRIVADA PROSEME	INTA Barrow
135	Co 1937 ^●^	ITM	nr	COLOSSEO/LÍNEA PRIVADA PROSEME	INTA Barrow
136	Capeiti ^●^	ITT	1940	CAPPELLI/EITI	INTA Marcos Juárez
137	Maristella ^●^	ITT	1969	DAUNO III/CAPEITI 8	INTA Marcos Juárez
138	Appullo ^●^	ITT	1973	CAPPELLI/GRIFONI//CAPEITI 8	INTA Barrow
139	Creso ^●^	ITT	1974	YAKTANA-54//NORIN-10/BREVOR/3/2*CAPELLI-63/4/3*TEHUACAN-60/5/CAPELLI B-144	INTA Marcos Juárez
140	Granato ^●^	ITT	1974	CAPPELLI/MARA-ITA//CAPPELLI	INTA Marcos Juárez
141	Gerardo 575 ^●^	ITT	1974	GIORGIO//CAPELLI/YUMA	INTA Marcos Juárez
142	Polesine ^●^	ITT	1975	FORLANI/AZIZIAH	INTA Marcos Juárez
143	Gabbiano ^●^	ITT	1976	CAPELLI / CONTO-MARZOTTO	INTA Marcos Juárez
144	Gerardo 645 ^●^	ITT	1978	GIORGIO//CAPELLI/YUMA	INTA Marcos Juárez
145	Duilio ^●^	ITT	1984	CAPELLI//ANHIGA/FLAMINGO	A.C.A.
146	Adamello ^●^	ITT	1985	VALFORTE/TURKISH SELECTION	A.C.A.
147	Gerardo 610 ^●^	ITT	na	GIORGIO//CAPELLI/YUMA	INTA Marcos Juárez
148	Gerardo 574 ^●^	ITT	na	GIORGIO//CAPELLI/YUMA	INTA Marcos Juárez
149	ITA1 ^●^	ITT	nr	SEL. CERZOS GAB 125 AN	INTA Marcos Juárez
150	GAB 125 ^●^	ITT	nr	na	INTA Marcos Juárez
151	Kofa ^●^	USA	1990	DERIVED FROM ‘‘DICOCCUM ALPHA POP-85 S-1” POPULATION	UCDAVIS
152	UC1113 ^●^	USA	2006	CD52600 (KIFS//RSS/BD1419/3/MEXIS-CP/4/WAHAS/5/YAV79	UCDAVIS
153	DGE-1 ^●^	USA	2006	LANGDON/L. ELONGATUM//LANGDON)*1/LANGDON]*8	Buck Semillas
154	Langdon(Dic-3A)-10 ^●^	USA	nr	LDN240/KHAPLI//LANGDON 308///MINDUM*3/VERNAL/4/VERNAL EMMER/3*MINDUM	Buck Semillas
155	Etit 38 ^●^	WAN	1963	ISRAELI LAND VARIETY	INTA Marcos Juárez
156	Omguer 4 ^●^	WAN	1983	GGOVZ355/GS//MEXI75	INTA Marcos Juárez
157	Cham 1 = Waha ^●^	WAN	1984	PLC"S"/RUF"S"/2/GTA"S"/RTTE	INTA Marcos Juárez
158	Wadalmez-1 ^●^	WAN	1985	GDOVZ 512/CIT/2/RUFF/FG/3/DWL 5023	INTA Marcos Juárez
159	Om Rabi ^●^	WAN	1985	JO/HAURANI = HAURANI X JORI-C69	INTA Marcos Juárez
160	Bilik No2 ^●^	WAN	1987	CR/STK	INTA Marcos Juárez
161	Korifla = Cham 3 ^●^	WAN	1987	DS15/GEIER	INTA Marcos Juárez
162	Haurani ^●^	WAN	1988	LOCAL LANDRACE SELECTION FROM SYRIA	INTA Marcos Juárez
163	Om Rabi 6^●^	WAN	1992	JO/HAURANI = HAURANI X JORI-C69	INTA Marcos Juárez
164	Om Rabi 5 ^●^	WAN	1993	JO/HAURANI = HAURANI X JORI-C69	INTA Marcos Juárez
165	Om Rabi 3 = Cham 5 ^●^	WAN	1993	JO/HAURANI = HAURANI X JORI-C69	INTA Marcos Juárez
166	Marrout ^●^	WAN	1997	GD/PEL-73081//CANDO/YAVARO-79	INTA Marcos Juárez
167	Bha ^●^	WAN	na	na	INTA Marcos Juárez
168	Heider//Mt/Ho ^●^	WAN	nr	HEIDER//MT/HO	INTA Marcos Juárez

^●^ Genotypes present in the subset of 119 accessions

^a^ Accessions are coded as ARM, modern Argentinian; ART, traditional Argentinian; CHI, Chile; CIM, CIMMYT; CYP, Cyprus; FRA, France; ITM, modern Italian; ITT, traditional Italian; USA, United States; WAN, West Asia North Africa region. Accessions from Argentina and Italy were divided into two groups according to the year of release (until and after 1985). Accessions labeled as "traditional" are those either bred or released until 1985.

^b^ na, not available; nr, not released.

^c^ Buck Semillas: Argentinian private company; INTA: Instituto Nacional de Tecnología Agropecuaria,Argentina; ACA: Asociación de Cooperativas Argentinas, Argentinian private company; ARI: Agricultural Research Institute (Cyprus); INIA: Instituto de Investigaciones Agropecuarias, Chile.

A field trial was carried out to purify all the accession in CEI-INTA Barrow, Argentina (38°20'S 60°13'W), in 2013. To this end, 5-m-long rows of each entry were sowed and off-type plants were eliminated. Seed from 1 to 2 selected plants were collected from each accession and maintained. For this field trial no special permission was required.

### Molecular analyses

Three-week-old seedlings grown from the purified seed were used for genomic DNA extraction following the protocol described by [33]. AFLP markers were assessed using the protocol described by [34] with some modifications, in an initial subset of 119 accessions (Table 1). Five hundred nanograms of genomic DNA were digested with *PstI* and *MseI* restriction enzymes for 3 hours at 37°C. Adapters of the known sequences *MseI* F (5´ GACGATGAGTCCTGAG 3´), *MseI* R (5´ TACTCAGGACTCAT 3´), *PstI* F (5´ CTCGTAGACTGCGTACATGCA 3´) and *PstI* R (5´ TGTACGCAGTCTAC 3´) were ligated to 10 μl of restricted DNA using T4-ligase (1U/μl) at 20°C during 3 hours. Pre-selective amplification was done using the adaptors P01 (5'GACTGCGTAGGTGCAGNNN 3') and M01 (5'GATGAGTCCTGAGTAANNN 3'). A 52 μl reaction mixture containing 2.5 μl of adaptor-ligated DNA was subjected to polymerase chain reaction (PCR) under the following conditions: 20 cycles of 94°C for 30 s, 56°C for 60 s, and 72°C for 60 s to finish at 4°C. The PCR product was diluted 6 times in TE buffer. Selective amplification was performed in a 25 μl reaction volume with 2 μl of diluted DNA as a template and considering six primer pair combinations (P40/M38, P40/M43, P41/M31, P41/M43, P41/M45 and P41/M39) (Table 2). The cycling conditions were performed in a two-step PCR program for a total of 40 cycles divided into 13 cycles of 94°C for 30 s, annealing at 65°C for 30 s decreasing 0.7°C per cycle and 72°C 60 s followed by 27 cycles of 94°C for 30 s, 56°C for 30 s, and 72°C for 60 s. The amplified products were separated by electrophoresis on 6.0% polyacrylamide gel and visualized by a silver staining protocol. Gels were scanned and stored in a computer to be analyzed. AFLP bands were scored in a dominant fashion either as present (1) or absent (0) by the registration of bands.

**Table 2:** AFLP oligonucleotide sequences used to analyze a durum wheat collection.

Primer	Code	Sequence
*Mse*AAA	M31	5´ GATGAGTCCTGAGTAAAAA 3´
*Mse*ACT	M38	5´ GATGAGTCCTGAGTAAACT 3´
*Mse*AGA	M39	5´ GATGAGTCCTGAGTAAAGA 3´
*Mse*ATA	M43	5´ GATGAGTCCTGAGTAAATA 3´
*Mse*ATG	M45	5´ GATGAGTCCTGAGTAAATG 3´
*Pst*AGC	P40	5´ GACTGCGTAGGTGCAGAGC 3´
*Pst*AGG	P41	5´ GACTGCGTAGGTGCAGAGG 3´

In a second analysis, a total of 85 SNPs were amplified using the KASP technology (https://www.lgcgroup.com) in the entire collection of 168 accessions, obtained after including more accessions to the original subset of 119. A touchdown PCR protocol was used starting with a 15 min hot enzyme activation at 94°C followed by 11 cycles of 94° for 30 s, 65°-55°C for 60 s (-0.8°C/cycle), 72°C for 30 sec and continued with 26 cycles of 94°C for 30 s, 57°C for 60 s, 72°C for 30 sec and a final step at 10°C. PCR was carried out arrayed in a 384 PCR plate and 5μl of PCR volume. The DNA samples were briefly centrifuged and oven dried at 60°C for 1 hour. SNP-specific KASP reagents, such as the Assay mix and the 2X KASP Master mix, including the fluorescent dyes FAM and VIC, were added to dried DNA samples (150 ng/well). Detailed protocols could be found in [35]. SNP markers were selected from CerealsDB (http://www.cerealsdb.uk.net) or developed by CIMMYT (S1 Table).Eighty-one markers were selected taking into account its putative map location on the A and B genomes. Four markers theoretically located on D genome were also tested for their specificity. The PCR amplified products were subjected to an end-point fluorescent reading using the PHERAstar Plus plate reader from BMG LABTECH. Alleles were assigned taking into account the differential fluorescent reading using excel software.

### Linkage disequilibrium

Linkage disequilibrium (LD) was tested to explore the suitability of the collection for genome-wide association mapping using the TASSEL v.3.0 [36]. Minor allele frequency (MAF) was calculated and LD analysis was performed without rare alleles (<5%). To avoid bias on LD calculation, polymorphic markers with residual heterozygosity or missing valued higher than 10% were removed from the data matrix. The LD in the collection was estimated for the SNP markers using the *r*^*2*^ index [37], which considers pairwise squared-allele frequency correlations. Pairwise LD values (*r*^*2*^) and their significance (P values) had been obtained by the two-sided Fisher's Exact test. In addition, LD (*r*^*2*^) was assessed on a subset of 119 accessions analyzed with both AFLP and SNP markers, calculated for the combination of both markers. Mapping positions were not available for the AFLP markers, and in the case of the SNP markers were mainly distributed at large genetic distances, according to CerealsDB website (http://www.cerealsdb.uk.net).

### Genetic relatedness among accessions and population structure

Population structure was analyzed using the clustering algorithm based on a Bayesian model [26, 38] implemented in the STRUCTURE v2.3.4 software (http://pritch.bsd.uchicago.edu/structure.html). Structure analysis was performed considering admixture as ancestry model with correlated allele frequencies [39]. Parameters were set at 100,000 burning periods and 100,000 Markov Chain Monte Carlo (MCMC) replicates using 10 independent runs for each number of subpopulations (*K* from 1 to 10). No prior information was provided regarding the pedigree or geographical origin of accessions to infer subpopulations. The true number of subpopulations (*K*) was calculated following the Evanno test [40] using the online platform STRUCTURE HARVESTER [41]. Accessions were assigned to a specific subpopulation when membership probability was ≥0.50. Population structure in the entire collection was investigated using SNP markers filtered with MAF ≥5% to minimize the bias effect of rare alleles [42]. Inferences in the subset of 119 accessions were performed using polymorphic AFLP and SNP markers (MAF ≥5% and <10% missing data).

Alternatively, a cluster analysis was carried out in the entire collection to determine the genetic relatedness among genotypes using a distance-based method. The Unweighted Pair Group Method with Arithmetic means (UPGMA) was carried out with Tassel 3.0 software using a modified Euclidean distance (https://bitbucket.org/tasseladmin/tassel-5-source/wiki/UserManual). In addition, the neighbor-joining (NJ) algorithm [43] was utilized based on a dissimilarity index calculated from the simple matching coefficient using DARwin software [44]. The NJ was implemented using 1,000 bootstrap replicates. Both dendrograms were drawn in the FigTree v1.4.3 software. The consistence of these two most common clustering algorithms was compared. Mantel test was performed to compare the genetic distances obtained [45]. The genetic relationships among accessions were also evaluated by Principal Coordinate Analysis (PCoA) using GenAlEx v6.5 software [46, 47]. Only the SNP markers with minor allele frequency (MAF) ≥5% were used in the PCoA.

Wright’s F-statistics (*Fst*) [48] was estimated in the entire collection. Nei’s genetic distance and identity [49] among subpopulations or origins were calculated using AFLP and SNP markers, and PCoA was carried out. In addition, an Analysis of Molecular Variance (AMOVA) was performed to assess variance among and within populations taking into account different geographical origins and genetic subpopulations determined by STRUCTURE software with the software package GenAlEx v6.5 using 999 permutations.

### Genetic diversity

AFLP and SNP markers were used to estimate genetic diversity parameters under the assumption that populations were in Hardy-Weinberg equilibrium (HWE), such as the percentage of polymorphic loci, effective number of alleles (*Ne*) per locus [37], heterozygosity observed (*Ho*), gene diversity (*He* = expected heterozygosity [50] also referred to as polymorphism information content (PIC) by [51], and Shannon’s information index (*I*) [52]. In the case of the subpopulations determined by structure analysis, total genetic diversity (*Ht*), genetic diversity within populations (*Hs*), number of private alleles (PA) and genetic differentiation coefficient among populations (*Gst* = *Ht*-*Hs*/*Ht*; [50]) were estimated. POPGENE V 1.32 software was used for the AFLP markers [53] while the GenAlex v6.5 software was used to analyze the SNP data. For the AFLP marker data, polymorphism information content (PIC) was also calculated by primer combination. Filtering by MAF was not applied for genetic diversity analyses, following the recommendations of [54], according to which MAF filtering had either very little or no effect on the results.

## Results

### AFLP genotyping

The analysis of the six AFLP primer pair combinations in the initial subset of 119 accessions yielded a total of 402 scorable loci. Of these loci, 182 (45.3%) were polymorphic. The total number of bands by primer pair ranged from 39 to 115 with an average of 67. The percentage of polymorphism ranged from 48.4% (P41M39) to 42.9% (P40M43) and the maximum number of scorable bands was detected using the primer pair P41M31 (*Pst*AGG/*Mse*AAA). The number of polymorphic bands ranged from 17 to 51, with an average of 30 polymorphic bands per primer combination (Table 3). The PIC evaluated as an average of each primer combination showed quite similar values with a mean value of 0.309. The primer pair P40M38 showed the lowest ability to detect polymorphisms (PIC = 0.276). A total of 125 and 108 AFLP loci were retained, to be used in the genetic diversity and population structure analyses, respectively (Table 3).

**Table 3:** Description of the total AFLP loci analyzed per primer combination.

AFLP primer combination	Polymorphic bands	Monomorphic bands	Total N° of bands	Markers for Structure^a^	Markers for GD^b^	Average PIC value ± SD^c^
P40M38	26	28	54	7	10	0.276(±0.181)
P40M43	30	40	70	21	25	0.339(±0.156)
P41M31	51	64	115	24	29	0.311(±0.172)
P41M43	27	33	60	15	17	0.306(±0.159)
P41M45	17	22	39	16	16	0.286(±0.121)
P41M39	31	33	64	25	28	0.334(±0.170)
Average	30	37	67	18	21	0.309
N° of bands	182	220	402	108	125	125

^a^ AFLP bands retained after filtering by MAF higher than 5% and missing data lower than 10%.

^b^ AFLP bands retained after filtering by missing data lower than 10%. GD, genetic diversity.

^c^ Polymorphism information content (PIC) calculated per primer combination and on average of all markers. SD, standard deviation.

### SNP genotyping

The KASP genotyping platform proved to be an effective discriminative method to obtain SNP marker data and to analyze the genetic diversity in our collection. Fifty six out of 85 SNP markers were polymorphic, 14 SNPs resulted to be monomorphic, 6 SNPs showed a high level of heterozygosity and 9 SNPs failed amplification in our collection (Tables 4 and S1). Among the mutations considered in this study, 52 corresponded to transitions and 33 to transversions. The high percentage of heterozygosity observed for some SNP markers (BS00020527, BS00012739, BS00012830, BS00013085, BS00077936, BS00003756, and BS00013985), ranging from 36.3% to 98.2%, could indicate a lack of specificity for the durum wheat genome.

**Table 4:** Number of KASP markers amplified in the durum wheat collection and their chromosomal position.

Chr. Arm	Total number	Polymorphic	Monomorphic	Heterozygotes	Failed	≥ 5% MAF
1AS	2	2	—	—	—	—
1AL	3	3	—	—	—	1
1BS	11	5	1	1	4	2
1BL	10	10	—	—	—	6
2AS	5	4	—	1		—
2BS	4	4	—	—	—	2
2BL	4	2	—	1	1	1
3AS	3	3	—	—	—	3
3AL	4	4	—	—	—	2
4AS	3	2	1	—	—	—
4AL	1	1	—	—	—	1
4BL	1	1	—	—	—	—
5AL	14	8	3	1	2	3
5BS	1	—	1	—	—	—
5BL	2	—	1	—	1	—
6AS	2	2	—	—	—	1
6BL	1	—	—	1	—	—
7AS	2	2	—	—	—	2
7AL	2	1	1	—	—	1
7BS	3	—	3	—	—	—
7BL	3	1	1	1	—	1
Total	81	55	12	6	8	26
Additional markers theoretically located in D genome						
2DS	1	1	—	—	—	—
2DL	1	—	—	—	1	—
3DS	1	—	1	—	—	—
5DL-1	1	—	1	—	—	—
D	4	1	2	—	1	—
Total	85	56	14	6	9	26

One SNP (BS00014897), which was reported to be located on 2DS (http://www.cerealsdb.uk.net/), resulted to be polymorphic in durum wheat, suggesting a wrong map position of this SNP or amplification in a homeologous chromosome in durum wheat. In addition, a second putative map location on 5BS is provided in this database. Our results showed that this SNP marker was polymorphic in the Italian cultivars Catervo and Granato. Seven out of the 56 polymorphic SNP markers were monomorphic in the subset of 119 accessions. On average, the number of missing data was low across polymorphic SNP with a maximum of 0.6% in two SNPs. SNP filtering by MAF resulted in 26 out of 56 polymorphic markers. Filtered markers were still equally distributed in the A (14) and B (12) genomes.

The KASP marker *Lr47-2* was originally designed for the leaf rust resistant gene *Lr47* based on the sequence PS10 (AJ238217) from [55]. Although it resulted not being diagnostic for leaf rust, it was highly polymorphic in our collection and was therefore included in our diversity analysis.

### Linkage disequilibrium estimates using AFLP and SNP markers

LD values were calculated in the subset of 119 accessions using AFLP and SNP markers in a combined analysis. To avoid the bias on the LD estimation, the analyses were carried out after MAF filtering. The estimated pairwise LD (*r*^*2*^) showed a very low number of significant *p* values, resulting in 4.9% of significant LD values (Table 5). The significant mean LD value (*r*^*2*^) was 0.11 while the total mean value was *r*^*2*^ *=* 0.016. A similar significant mean LD value was obtained in the entire collection using SNP (*r*^*2*^ = 0.12).

**Table 5:** Linkage disequilibrium (LD) estimates.

		AFLP+ SNP	
Number of accessions		119	
Number of markers		134	
Pairwise measurement ^a^		N	%
*r*^*2*^ <0.1		277	3.1
0.2> *r*^*2*^ >0.1		133	1.5
0.5> *r*^*2*^ >0.2		21	0.2
*r*^*2*^ >0.5		5	0.1
Total significant pairs^b^		436	4.9
Mean significant *r*^*2*^ ^c^		0.11	
Global average *r*^*2*^		0.016	
Total pairwise combinations		8911	100

^a^ Number of pairwise significant (*P* value <0.01) LD estimates according to the ranges of r^2^ values.

^b^ Number and percentage of total *r*^*2*^ estimates with *P* value <0.01.

^c^ Average *r*^*2*^ values calculated only using significant *P* value <0.01 pairwise estimates.

### Population structure in the entire collection

**Fig 1:**
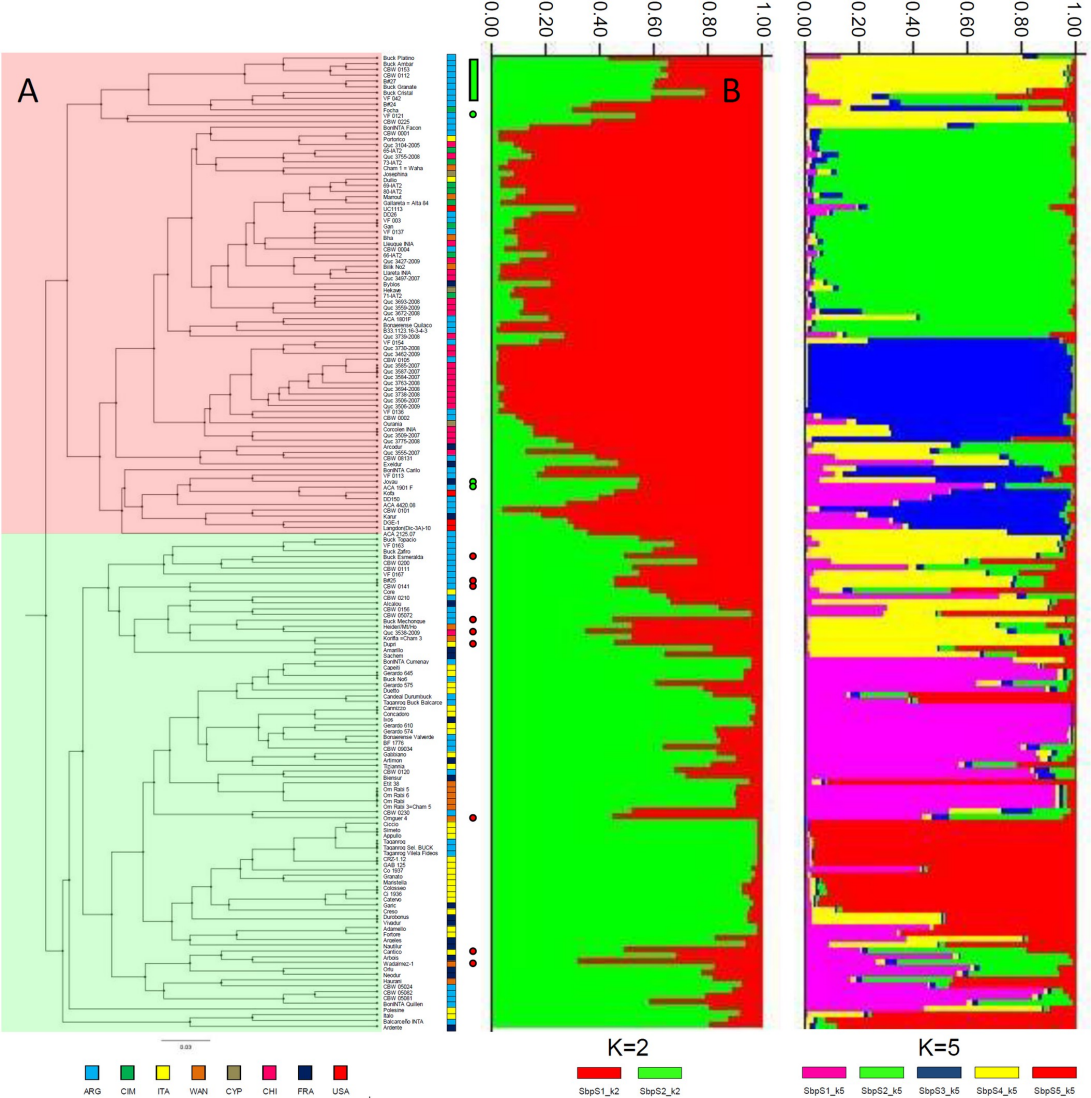
Comparison of population structure obtained by UPGMA cluster analysis and a Bayesian model (STRUCTURE) using SNP markers in the whole durum wheat collection. (A) Circles or bars colored in green and red indicates the accessions with differences in the subpopulation assigned by UPGMA and STRUCTURE analyses. The country of origin of accessions is indicated by colored squares in front of the name of accessions. (B) Results for *K* = 2 and *K* = 5 obtained by STRUCTURE.

Population structure was further explored in the entire collection composed of 168 accessions of different origins using 26 SNP selected markers and applying the Bayesian clustering method with STRUCTURE software (Fig 1). The maximum *ΔK* value was observed at *K* = 2, with a second peak at *K* = 5 (S1A Fig). Fig 1B shows the membership probability obtained at *K* = 2 and *K* = 5 for each genotype. Q matrix was calculated as an average of ten runs for *K* = 2 and *K* = 5 (S2A and S2B Table). According to a membership probability ≥ 0.5, 82 accessions (48.81%) were assigned to subpopulation 1 (SbpS_1) and 86 accessions (51.19%) to subpopulation 2 (SbpS_2) for *K* = 2.

**Fig 2:**
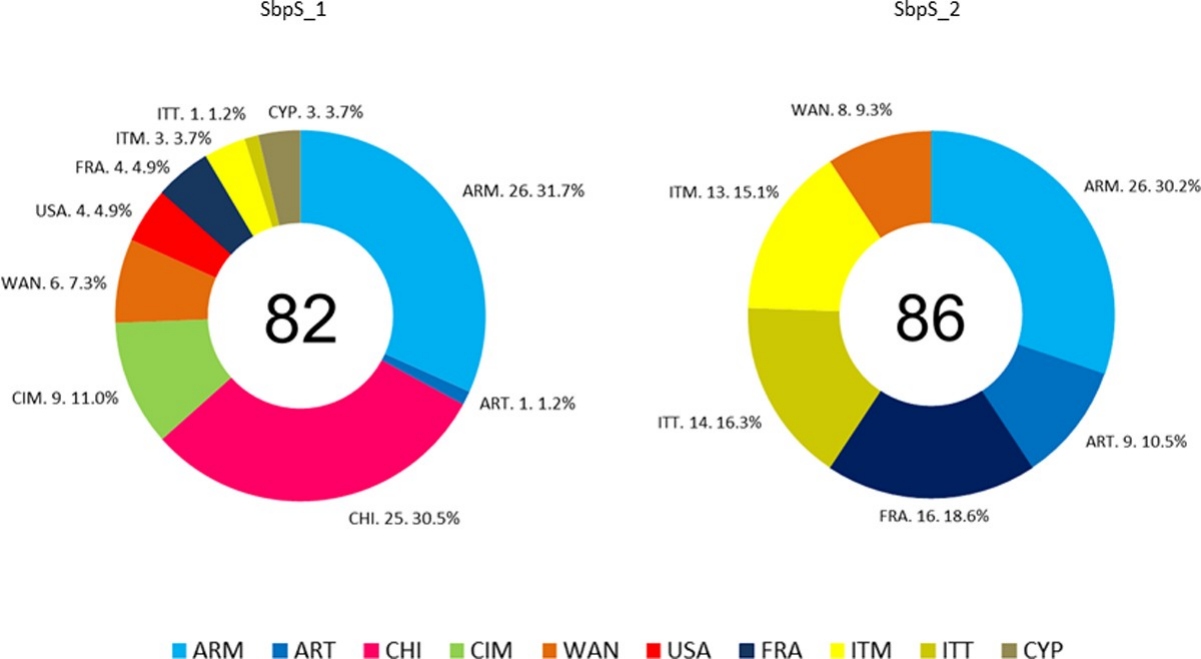
Ring graph showing the origin of accessions included in each subpopulation according to STRUCTURE analysis (*K* = 2, maximum) using 26 SNP. The accessions are coded as ARM, modern Argentinians; ART, traditional Argentinians; CHI, Chile; CIM, CIMMYT; CYP, Cyprus; FRA, France; ITM, modern Italians; ITT, traditional Italians; USA, United States; WAN, West Asia North Africa region. Accessions from Argentina and Italy were divided in two groups according to the year of release (until and after 1985). It was considered "traditional" accessions to those bred or released before or up to 1985.

The analysis of the origin of accessions in both subpopulations for *K* = 2 showed that SbpS_1 was mainly composed of germplasm from Argentina (mostly moderns [26] and one traditional) and Chile (25) with CIMMYT-derived pedigrees. All the Chilean accessions have CIMMYT ancestry (S2A Table). Also, all the CIMMYT accessions (9) obtained from INTA germplasm bank or international nurseries were assigned to this subpopulation. In addition, SbpS_1 also included germplasm from WANA region (6) and a small number of accessions from USA (4), France (4), Italy (4), and Cyprus (3) (Fig 2). The composition analysis in the pedigrees of SbpS_1 accessions revealed several representative genotypes from CIMMYT, such as Altar 84, Yavaros 79, Mexicali 75, Flamingo, Altar84-derived as Aconchi 89 and the Plata group. Founder genotypes from North-Dakota (USA), such as Lakota, Cando and Langdon-derived contributed to the pedigree in this subpopulation, in particular in the modern Argentinian germplasm. All the accessions from USA, such as UC1113, Kofa, DGE-1 and Langdon (Dic-3A)-10, were assigned to SbpS_1.

On the other hand, Sbp_2 for *K* = 2 was mainly composed of accessions from Argentina (moderns [26] and traditionals [9]), Italy (moderns [13] and traditionals [14]), France (16) and WANA region (8) (Fig 2). The number of accessions from these four origins was higher in SbpS_2 than in SbpS_1. The traditional Argentinian genotypes were mostly included in this subpopulation. The SbpS_2 can, in general, be considered either as germplasm with Mediterranean basin origin or as Argentinian genotypes with parental lines or ancestry from this region, the Italian germplasm being the main contributor. The analysis of Argentinian cultivar pedigrees or breeding lines included in SbpS_2 revealed that 17 of 35 genotypes evidenced a strong contribution of Italian germplasm, and that most of the remaining materials were CIMMYT-derived genotypes with Italian ancestors, such as Cappelli or the Gerardo group. The germplasm included in the Gerardo group corresponded to selections of the cross GIORGIO//CAPELLI/YUMA obtained by [56] in Italy.

**Fig 3:**
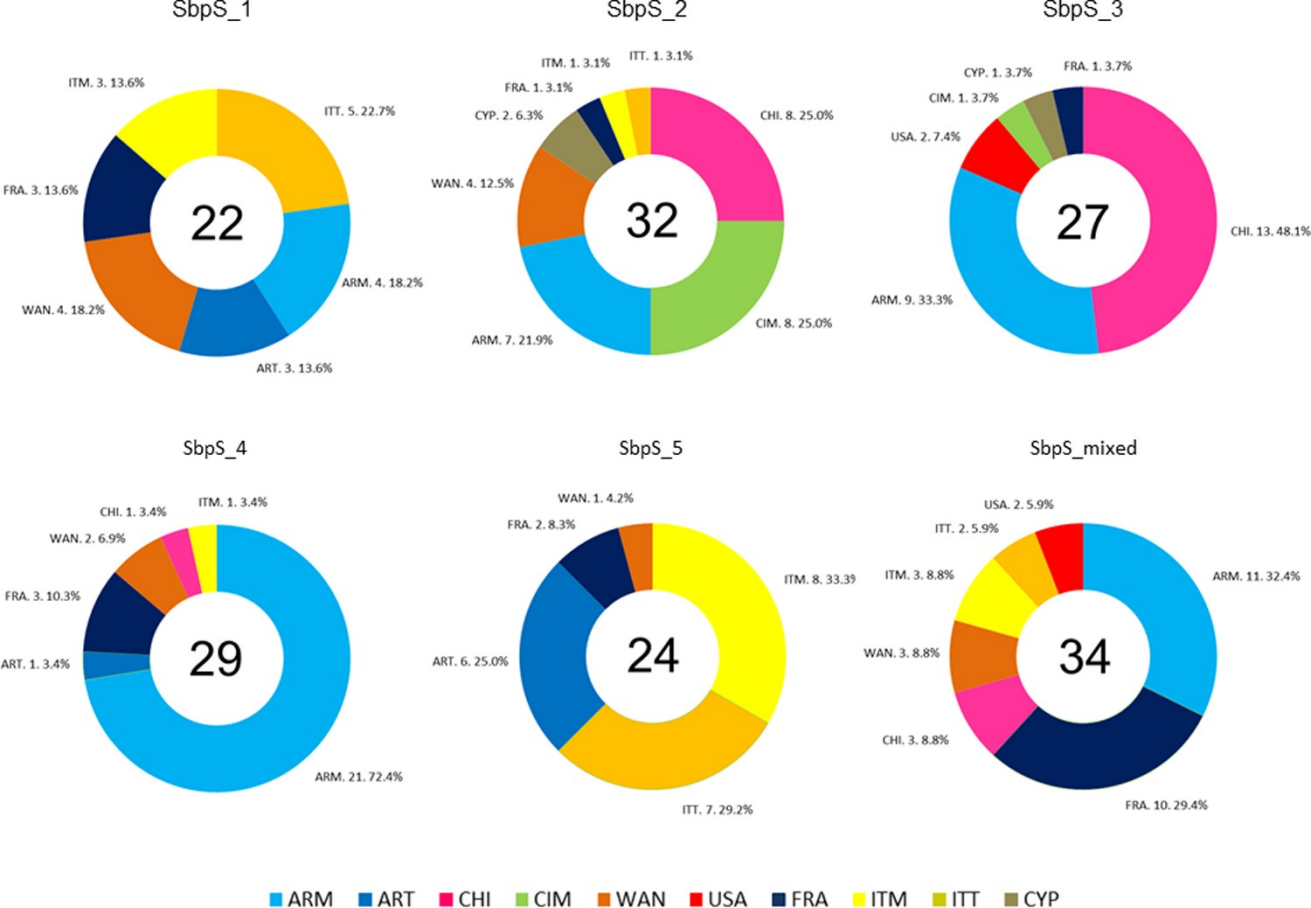
Composition of each subpopulation (*K* = 5) according to the origin of accessions. The accessions are coded as ARM, modern Argentinians; ART, traditional Argentinians; CHI, Chile; CIM, CIMMYT; CYP, Cyprus; FRA, France; ITM, modern Italians; ITT, traditional Italians; USA, United States; WAN, West Asia North Africa region.

The second minor peak which was observed in the *ΔK* plot at *K* = 5 (S1A Fig) and which was detected using SNP markers (SbpS), was taken into account to analyze the substructure in our durum wheat collection (S2B Table). Each accession was assigned to the subpopulations with a membership probability of 0.5. For *K* = 5, five subpopulations were detected and one additional group including 34 accessions with admixture ancestry (Fig 3). The SbpS_1 for *K* = 5 included modern and traditional Argentinian and Italian genotypes (15), genotypes from France (3) and the Om Rabi sister lines (4) from WANA region. One characteristic of this subpopulation was the presence of five genotypes with pedigree from the Gerardo group and three breeding lines with Gerardo group genotypes as parental line.

The SbpS_2 for *K* = 5 was composed of accessions from CIMMYT (8), Chile (8), modern Argentinian germplasm (7), WANA region (4), Cyprus (2), and Italy (2), all carrying mainly a CIMMYT-derived pedigree. The SbpS_3 corresponded to a second group with a CIMMYT-derived pedigree, including genotypes from Chile (13), modern Argentinian germplasm (9), CIMMYT (1), Cyprus (1), France (1) and two Langdon-derived materials from USA.

Moreover, the SbpS_4 for *K* = 5 was mainly composed of modern Argentinian germplasm (21), followed by French (3) and WANA (2) accessions and three additional accessions. The pedigree analysis showed a prevalence of CIMMYT germplasm as well as some Italian genotypes, such as Belfugitto, Farro Lunga and the Gerardo group. The presence of a Gerardo group-derived line, Bonaerense Valverde (selection 516), was also identified in the pedigree of some Argentinian breeding lines.

The subpopulation 5 (SbpS_5) in the *K* = 5 model was composed of modern (8) and traditional (7) Italian accessions, traditional Argentinian germplasm (6), and accessions from France (2) and from WANA region (1) (Fig 3). All Argentinian tall genotypes derived from Taganrog were included in this group. The mixed population with a membership probability below 0.5 threshold included accessions from Argentina (modern germplasm [11]), France (10), Chile (3), WANA region (3), Italy (modern germplasm [3], traditional germplasm [2]), and USA (2).

### AFLP and SNP markers to assess population structure in the subset of 119 accessions

The subset of 119 accessions genotyped with AFLP and SNP markers was used to analyze the population structure and the suitability of each type of marker to establish the number of subpopulations. A model-based Bayesian cluster analysis with STRUCTURE software was performed using ─separately─ 26 SNP markers and 108 AFLP polymorphic bands (treated as recessive allele). As for the entire collection, the SNP marker analysis identified two subpopulations by means of the *ΔK* parameter obtained by the method proposed by [40] (S1B Fig). A detailed description of the subpopulations obtained using SNPs was performed before when the entire collection was considered. However, when the population structure analysis was performed using AFLP markers, the maximum *ΔK* being obtained at *K* = 6 according to the *ad hoc* Evanno test. The *ΔK* calculated at *K* = 7 was slightly lower than that at *K* = 6 and a second minor peak was detected at *K* = 3, thus suggesting a possible stratification in three initial groups and 6–7 genetically closest subpopulations (S1C Fig). The membership probability (Q matrix) of each accession to each subpopulation for the *K* = 6 model was obtained as an average of ten runs and is shown in S3 Table.

Regarding to the subpopulations obtained with AFLP (SbpA) for the maximum *ΔK* (*K* = 6), the subpopulation 1 (SbpA_1) comprised only Argentinian and Italian modern germplasm and the subpopulations SbpA_2 and SbpA_4, both were mainly composed of traditional Italian and Argentinian germplasm. The SbpA_2 also included germplasm from WANA (2), whereas SbpA_4 also comprised germplasm from WANA (1), France (2) and modern Argentinian genotypes (3). Furthermore, while most of the accessions in SbpA_2 corresponded to tall genotypes, in SbpA_4 only six of 20 were tall genotypes.

The SbpA_3 was composed mainly of germplasm from WANA region (8) while SbpA_5 mostly integrated of French accessions and six additional accessions from other origins, such as CIMMYT (1), USA (1), Italy (1), Argentina (1) and WANA region (2). SbpA_6 was the largest subpopulation identified by AFLP using a *K* = 6 model and composed mainly of Argentinian modern (18) accessions followed by CIMMYT (6) genotypes and a few accessions from Italy (4), USA (2) and WANA (1). A characteristic of this subpopulation was the predominance of CIMMYT-derived germplasm. Moreover, twelve accessions were not assigned to any specific subpopulation and they were considered as a mixed group. Surprisingly, Altar 84 = Gallareta, which is considered to be a founder genotype, was part of this mixed group with prevalence of membership for SbpA_3 and SbpA_6.

### Hierarchical clustering of the entire collection

Population structure in the durum wheat collection was also investigated with distance-based methods using the 26 selected SNP markers. Cluster analyses were performed using Unweighted Pair Group Method with Arithmetic means (UPGMA) and neighbor-joining (NJ) algorithm and the results collected were further compared (S2 Fig). The Mantel test performed between the genetic distance calculated by Darwin and TASSEL softwares indicated a correlation of r² = 0.994. Both clustering methods -UPGMA and NJ- showed their ability to cluster sister lines as for example Om Rabi group, the Gerardo group, BonInta Quillen and their sister lines, and Buck Granate and B#27. Related accessions, such as CIMMYT lines (IAT2) and several Chilean breeding lines (QUC), were also clustered together. UPGMA and NJ clustering methods could associate parental lines and their progeny, such as the cultivar Kofa and derivative genotypes, Taganrog, their selections or derivative cultivars, Buck Topacio and Buck Zafiro, BonInta Cumenay with their parental line Taganrog Buck Balcarce.

However, when both types of dendrograms were compared with the results obtained with the STRUCTURE software at a maximum *ΔK* = 2, UPGMA clustering method showed the highest agreement (Fig 1). Compared to the Bayesian method, the UPGMA clustering method identified 2 main groups and only 10 differences in SbpS_1 and 9 differences in SbpS_2.

### Principal Coordinates Analysis (PCoA)

The genetic relationships among genotypes in the entire collection were also investigated through PCoA with the 26 SNP selected markers to test the best genotype assignation to each subpopulation. The accessions were colored in the PCoA plot according to their membership to the subpopulations defined by STRUCTURE software for *K* = 2 and *K* = 5 (S3A and S3B Fig). The comparison of the results derived from PCoA and STRUCTURE software analyses performed for *K* = 2 in order to assign genotypes to each subpopulation revealed a high coincidence with the exception of only 3 accessions that were grouped differently by both methods. The accessions were clustered in the PCoA plot into two groups corresponding to SbpS_1 and SbpS_2, in the *K* = 2 model, for the accessions located to the right and to the left of the vertical axis, respectively. The percentage of variance explained by the first three axes was 38.7% (S4A Table). Although the results derived from the PCoA analysis performed for the *K* = 5 model agreed in general with the subpopulations assigned by the STRUCTURE software analysis, either more differences in the subpopulation assignment or subpopulation overlapping were detected. The comparison of the three methods applied showed that 72 accessions were clearly assigned to SbpS_1 and 75 to SbpS_2 (S5 Table).

The differences observed at subpopulation level for *K* = 5 (S4B Table and S3C Fig) through PCoA explained 95.1% of variance when the first 3 axes were considered (S4C Table). The subpopulations SbpS_2 and SbpS_3 were clustered together, which agreed with the prevalence of CIMMYT-derived pedigrees. The subpopulation SbpS_1, which was mainly represented by Mediterranean or Mediterranean-derived germplasm, and the subpopulation SbpS_5, mainly composed of the traditional Italian/Argentinian accessions, were plotted separately from the modern Argentinian germplasm population (SbpS_4).On the other hand, the PCoA analysis performed taking into account the origin of accessions revealed that either cultivars or breeding lines from Cyprus, Chile and CIMMYT were highly related to each other (S4D Table and S3D Fig).

Otherwise, the similarities among accession´s origins (8) or the genetic subpopulations at *K* = 6, calculated with 108 AFLP markers, were also explored via PCoA based on Nei´s genetic distances in the subset of 119 accessions (S6A and S6C Table and S4 Fig). Six main geographical origins totalizing 8 groups were considered. The Italian and Argentinian genotypes were divided between traditional and modern accessions taking into account the history of the process of Argentinian breeding programs. The PCoA analysis carried out based on the origins of accession showed that the Argentinian and Italian traditional genotypes were closely related whereas the modern Argentinian accessions were plotted between CIMMYT and Italian modern germplasm but in the same quadrant as that of CIMMYT and USA accessions. In addition, the genotypes from WANA region and France were observed to be closely related to each other but less related to the Argentinian accessions (S4B Fig).

Although AFLP markers showed that the traditional Italian and Argentinian genotypes were genetically related, the relationship between modern and traditional Italian materials evidenced by SNP markers was stronger than that shown by AFLP markers (S3B and S4B Figs). The genetic distance calculated with SNP markers among the modern Argentinian accessions and the germplasm from France and WANA region was lower than that calculated with AFLP markers. Likewise, the genetic distance calculated with SNP markers between Argentinian and USA genotypes was higher than that calculated with AFLP markers.

Moreover, the PCoA analysis based on the genetic subpopulations (*K* = 6) obtained with AFLP showed that the subpopulations SbpA_2, SbpA_4 and SbpA_5 were genetically more distant. Two of them (SbpA_2, SbpA_4) were mainly composed of traditional Argentinian and Italian germplasm whereas Sbp_5 included a high proportion of French germplasm. In contrast, SbpA_1, represented by modern Argentinian and Italian genotypes, and SbpA_mixed, which also included modern Argentinian and Italian genotypes, were observed to be more related to each other. SbpA_3 and SbpA_6, which included accessions mainly from WANA region and CIMMYT-derived genotypes, respectively, were plotted together. PCoA evidenced small genetic differences for these last four groups (S4A Fig).

### Analysis of molecular variance based on accession origins and genetic subpopulations

The percentage of variance explained among and within the different geographical origins and genetic subpopulations using AFLP markers for a subset of 119 accessions and SNP markers in our entire durum wheat collection was calculated by means of an analysis of molecular variance (AMOVA) test based on *PhiPT* index. All analyses were highly significant (p<0.001). In both cases, the AMOVA test which considered geographical origins explained the lower percentage of variance among groups −9% for AFLP and 16% for SNP markers (S5 Fig)–compared to the percentage of variance when subpopulations were determined by the STRUCTURE software −19% for AFLP and 33% for SNP markers–. The remaining variance was explained by the accessions within groups (origins or subpopulation). Despite its smaller number, the SNP markers explained higher percentage of variance between genetic subpopulations determined by STRUCTURE than the AFLP markers.

### Genetic diversity

A total of 56 SNP were used in order to evaluate the genetic diversity in the entire collection and 125 AFLP and 56 SNP were used with the same purpose in a subset of 119 accessions. In the entire collection, it was found that the Italian accessions Granato and Maristella, and the Chilean breeding line QUC 3506–2009 were the ones with the highest number of rare alleles. As a measure of the level of polymorphism, several descriptive indices were used, such as the effective number of alleles (*Ne*), Nei's gene diversity (*He*) also referred to as heterocigozity or PIC, Shannon's Information index (*I*) or the coefficient of genetic differentiation among subpopulations (*Gst*). Genetic diversity index was estimated per locus and also per subpopulation taking into account either the geographical origin or genetic subpopulation.

The genetic diversity results estimated per locus in the entire collection using 56 polymorphic SNP are shown in Table 6. A heterozygosity (*He*) mean value of 0.183 and a coefficient of genetic differentiation (*Gst*) value of 0.139 were obtained. Considering the *K* = 5 model of STRUCTURE, *He* values were higher in SbpS_mixed followed by Sbp_4 (Table 7). The coefficient of genetic differentiation among subpopulations (*Fst*) was calculated for *K* = 5 and the main differences were found between the Sbp_5 (mostly old material) and Sbp_3 (CIMMYT-derived germplasm) (S7A Table). Considering the geographical origins of the complete collection, the traditional Italian and modern Argentinian genotypes followed by French accessions were found to exhibit the highest genetic variance for all indices. The highest genetic differences among origins were found between traditional Argentinian/Italian germplasm and the Cyprus accessions (S7B and S7C Table).

**Table 6:** Allele frequencies and genetic diversity indices estimated per locus using 56 SNP markers in a durum wheat collection of 168 accessions.

SNP ID ^a^	*N*	SNP type	f(1)	f(2)	*Ne*	*Ho*	*He*	*I*	*Hs*	*Ht*	*Gst*
**BS00003575**	168	C/T	0.911	0.089	1.194	0.000	0.163	0.301	0.163	0.163	-0.003
BS00003634	168	C/T	0.994	0.006	1.012	0.000	0.012	0.036	0.012	0.012	0.000
BS00003776	168	C/T	0.006	0.994	1.012	0.000	0.012	0.036	0.012	0.012	0.000
BS00003807	168	A/G	0.006	0.994	1.012	0.000	0.012	0.036	0.012	0.012	0.000
BS00004129	168	G/C	0.949	0.051	1.106	0.006	0.096	0.200	0.096	0.096	-0.002
**BS00004158**	167	C/T	0.353	0.647	1.841	0.000	0.457	0.649	0.379	0.456	0.169
**BS00004224**	168	C/T	0.446	0.554	1.977	0.000	0.494	0.687	0.316	0.498	0.364
BS00004546	168	A/T	0.994	0.006	1.012	0.000	0.012	0.036	0.012	0.012	0.000
BS00004673	168	G/T	0.021	0.979	1.043	0.018	0.041	0.101	0.040	0.040	0.006
BS00004727	168	A/G	0.994	0.006	1.012	0.000	0.012	0.036	0.012	0.012	0.000
BS00005036	168	A/G	0.006	0.994	1.012	0.000	0.012	0.036	0.012	0.012	0.000
**BS00005060**	168	C/T	0.179	0.821	1.415	0.000	0.293	0.469	0.284	0.298	0.046
BS00005092	168	A/C	0.994	0.006	1.012	0.000	0.012	0.036	0.012	0.012	0.000
**BS00005117**	168	G/C	0.649	0.351	1.837	0.000	0.456	0.648	0.377	0.455	0.171
BS00005272	168	C/T	0.024	0.976	1.049	0.000	0.046	0.113	0.045	0.046	0.018
**BS00005311**	168	A/G	0.917	0.083	1.180	0.000	0.153	0.287	0.152	0.156	0.026
**BS00005343**	168	C/T	0.345	0.655	1.825	0.000	0.452	0.644	0.358	0.451	0.206
**BS00009274**	168	G/C	0.625	0.375	1.882	0.000	0.469	0.662	0.309	0.467	0.339
**BS00009848**	168	G/C	0.601	0.399	1.921	0.000	0.480	0.673	0.403	0.480	0.160
**BS00010779**	168	G/C	0.176	0.824	1.408	0.006	0.290	0.465	0.241	0.296	0.186
**BS00010888**	168	G/C	0.824	0.176	1.408	0.006	0.290	0.465	0.241	0.296	0.186
**BS00012056**	168	A/G	0.518	0.482	1.997	0.000	0.499	0.693	0.332	0.501	0.337
**BS00012587**	168	A/G	0.491	0.509	1.999	0.006	0.500	0.693	0.493	0.503	0.020
**BS00012743**	168	G/C	0.494	0.506	2.000	0.000	0.500	0.693	0.480	0.503	0.045
**BS00012772**	168	A/G	0.589	0.411	1.938	0.012	0.484	0.677	0.408	0.485	0.158
BS00014046	168	G/C	0.033	0.967	1.068	0.006	0.063	0.144	0.061	0.062	0.027
BS00014101	168	A/C	0.967	0.033	1.068	0.006	0.063	0.144	0.061	0.062	0.027
**BS00014199**	168	G/C	0.827	0.173	1.400	0.000	0.286	0.460	0.288	0.288	0.002
BS00014413	168	A/G	0.952	0.048	1.100	0.012	0.091	0.191	0.092	0.091	-0.006
BS00014897	168	A/C	0.012	0.988	1.024	0.000	0.024	0.065	0.023	0.023	0.006
BS00014923	168	A/G	0.985	0.015	1.030	0.006	0.029	0.077	0.029	0.029	0.009
**BS00015223**	168	A/G	0.905	0.095	1.208	0.000	0.172	0.314	0.174	0.173	-0.006
**BS00015274**	168	C/T	0.679	0.321	1.774	0.000	0.436	0.628	0.422	0.440	0.042
**BS00016097**	168	G/C	0.241	0.759	1.577	0.006	0.366	0.552	0.330	0.365	0.093
BS00016725	168	A/C	0.994	0.006	1.012	0.000	0.012	0.036	0.012	0.012	0.000
**BS00018086**	168	C/T	0.75	0.25	1.600	0.000	0.375	0.562	0.375	0.379	0.009
BS00018367	168	A/C	0.994	0.006	1.012	0.000	0.012	0.036	0.012	0.012	0.000
**BS00018474**	168	C/T	0.06	0.94	1.126	0.000	0.112	0.226	0.107	0.111	0.033
BS00020741	168	G/C	0.006	0.994	1.012	0.000	0.012	0.036	0.012	0.012	0.000
BS00003694	168	C/T	0.03	0.97	1.061	0.000	0.058	0.134	0.057	0.057	0.004
BS00003837	168	C/T	0.994	0.006	1.012	0.000	0.012	0.036	0.012	0.012	0.000
BS00004378	168	A/G	0.012	0.988	1.024	0.000	0.024	0.065	0.024	0.024	-0.006
BS00019332	168	A/G	0.988	0.012	1.024	0.000	0.024	0.065	0.024	0.024	-0.006
**Lr47-2**	168	A/G	0.536	0.464	1.990	0.000	0.497	0.691	0.296	0.500	0.408
BS00022093	168	G/C	0.012	0.988	1.024	0.000	0.024	0.065	0.024	0.024	-0.006
BS00003743	168	C/T	0.949	0.051	1.106	0.006	0.096	0.200	0.097	0.097	-0.005
BS00022851	168	A/G	0.932	0.068	1.146	0.042	0.128	0.250	0.119	0.126	0.054
**BS00023148**	167	C/T	0.144	0.856	1.326	0.036	0.246	0.412	0.249	0.248	-0.004
**BS00108257**	168	C/T	0.083	0.917	1.180	0.000	0.153	0.287	0.142	0.151	0.058
**BS00077329**	168	G/C	0.065	0.935	1.139	0.012	0.122	0.242	0.119	0.121	0.021
**BS00022411**	168	A/C	0.696	0.304	1.733	0.000	0.423	0.614	0.342	0.421	0.187
BS00082002	168	C/T	0.012	0.988	1.024	0.000	0.024	0.065	0.023	0.023	0.006
BS00094343	168	A/G	0.988	0.012	1.024	0.000	0.024	0.065	0.023	0.023	0.006
BS00066143	168	C/T	0.976	0.024	1.049	0.012	0.046	0.113	0.046	0.046	0.008
BS00010757	168	A/T	0.982	0.018	1.036	0.000	0.035	0.090	0.036	0.036	-0.004
BS00075379	168	G/T	0.988	0.012	1.024	0.000	0.024	0.065	0.023	0.023	0.006
Min. ^j^	168				1.012	0.000	0.012	0.036	0.012	0.012	-0.006
Max.	168				2.000	0.042	0.500	0.693	0.493	0.503	0.408
Mean	168				1.304	0.004	0.183	0.291	0.158	0.184	0.139
S.E.					0.048	0.001	0.025	0.034	0.020	0.025	0.026

*N*, number of samples; f(1), Allele frequency of the 1st allele indicated in the SNP type; f(2), Allele frequency of the 2cd allele indicated in the SNP type; *Ne*, Effective number of alleles; *Ho*, observed heterozigosity; *He*, Nei's gene diversity or heterozigosity; *I*, Shannon's Information index; *Ht*, total genetic diversity; *Hs*, genetic diversity within populations; *Gst*, coefficient of genetic differentiation among subpopulations calculated based on *K* = 2 (maximum *ΔK*); Min, minimum value; Max, maximum value; S.E., standard error.

^a^ Markers in bold font correspond to the SNP markers selected by MAF.

**Table 7:** Genetic diversity among subpopulations assessed using 56 SNP markers in a durum wheat collection of 168 accessions.

Maximum *ΔK* (*K* = 2)								
**Subpopulation**	***N***	***Ne***	***Ho***	***He***	***I***	**n° PL**	**% PL**	
SbpS_1	82	1.217 (±0.038)	0.002 (±0.001)	0.141 (±0.022)	0.231 (±0.031)	45	80.4	
SbpS_2	86	1.275 (±0.045)	0.005 (±0.002)	0.171 (±0.023)	0.276 (±0.032)	48	85.7	
Total population	168	1.304 (±0.048)	0.004 (±0.001)	0.183 (±0.025)	0.291 (±0.034)	56	100.0	
2nd *ΔK* peak (*K* = 5)								
**Subpopulation**	***N***	***Ne***	***Ho***	***He***	***I***	**n° PL**	**% PL**	**n° PA**
SbpS_1	22	1.153 (±0.035)	0.003 (±0.03)	0.099 (±0.002)	0.158 (±0.02)	22.0	39.3	.
SbpS_2	32	1.14 (±0.034)	0.002 (±0.03)	0.091 (±0.001)	0.146 (±0.02)	22.0	39.3	.
SbpS_3	27	1.171 (±0.033)	0.001 (±0.029)	0.117 (±0.001)	0.196 (±0.019)	33.0	58.9	8
SbpS_4	29	1.249 (±0.046)	0.007 (±0.035)	0.149 (±0.003)	0.231 (±0.025)	32.0	57.1	.
SbpS_5	24	1.205 (±0.033)	0.004 (±0.03)	0.140 (±0.002)	0.231 (±0.02)	35.0	62.5	4
SbpS_mixed	34	1.287 (±0.047)	0.004 (±0.034)	0.175 (±0.002)	0.276 (±0.024)	40.0	71.4	1
Total	168							

*N*, number of samples; *Ne*, Effective number of alleles; *Ho*, Observed heterozigosity; *He*, Nei's gene diversity or heterozigosity; *I*, Shannon's Information index; n° PL, Number of polymorphic loci; % PL, Percentage of polymorphic loci; n° PA, number of private alleles.

The subset of 119 accessions was used to compare the genetic diversity assessed by AFLP and SNP markers. Only 49 of 56 SNP markers (87.5%) and all the selected AFLP markers were found to be polymorphic in this subset (S8 Table). AFLP markers proved to have a higher capacity than the SNPs to capture genetic variation in our subset of genotypes, obtaining in all cases higher index values (Table 8). The mean *Gst* value obtained using AFLP markers was higher, thus showing that this analysis was also more powerful to discriminate subpopulations.

**Table 8:** Genetic diversity mean values obtained with each type of marker for *K* = 2 in the subset of 119 accessions.

Marker	*N*	*Ne*	*He*	*I*	*Hs*	*Ht*	*Gst*
SNP	119	1.303	0.182	0.289	0.160	0.183	0.131
AFLP	119	1.604	0.352	0.524	0.339	0.262	0.225

*N*, number of accessions; *Ne*, Effective number of alleles; *He*, Nei's gene diversity or heterozigosity; *I*, Shannon's Information index; *Ht*, Total genetic diversity; *Hs*, Genetic diversity within populations; *Gst*, Coefficient of genetic differentiation among populations calculated at the maximum *ΔK*.

Our analysis of genetic diversity considering of subpopulations detected by STRUCTURE software in this subset showed that genetic variability measured as *He* was higher in SbpA_mixed, SbpA_6 and SbpA_1 (S8B Table). SbpA_6 corresponded to the subpopulation which included mostly modern Argentinian genotypes. Taking into account the origin of accessions, the traditional Italian genotypes followed by the modern Argentinian accessions evidenced the highest genetic variance estimated by AFLP markers (S8C Table).

## Discussion

### Genetic characterization

Our study was aimed at characterizing the level of polymorphism in a durum wheat collection based on SNP and AFLP markers. Our results proved that both marker systems were informative providing complementary data that helped to describe the germplasm, its genetic origin and its diversity level. Although AFLP markers are at present considered an old marker system they proved to be an efficient strategy not only to perform genetic fingerprinting in durum wheat but also to establish genetic relationships among accessions. Further new alternatives to use AFLP markers, such as the use of fluorescently labeled primers, have been proposed [57].

AFLP markers have been extensively used to detect DNA polymorphisms among durum wheat cultivars from different regions [1, 3, 10, 58–60]. In contrast, the use of SNP markers to measure variance in a genetic background is a more recent strategy and it is in general based on either array technologies [61] or on the development of specific genes [62]. Still, SNP markers are less frequently used to characterize germplasm collections [63–64].

Both markers showed a good level of polymorphism (AFLP markers-45.3%-, SNP -65.9%-), as was previously reported by [64] with 69.1% of polymorphic SNP markers in cultivated wheat or by [63] who reported 75.5% of polymorphic SNP loci. As to AFLP markers, an average of 13.3% of polymorphic fragments was reported by [65] whereas other authors detected 31% [5], 48.7% [1] and 64% [59] with a variable number of accessions and primer combinations. A higher number of rare alleles were observed in the SNP set with respect to the AFLP´s one, which showed only 13.6% of infrequent alleles.

No previous KASP marker analyses have been performed to date to explore genetic background diversity in durum wheat. The present study is, in fact, the first wide molecular characterization of the Argentinian durum wheat germplasm. Most of the SNP markers (18 of 26) selected after MAF filtering and used to estimate genetic relationships were not included in the 35K array of Affymetrix and presented a MAF average of 32.2%. KASP is an endpoint genotyping technology with several advantages, such as simplicity, cost-effectiveness and flexibility to determine both SNP and insertion/deletion genotypes [11].

### Linkage disequilibrium

Both the ability to capture significant associations among polymorphic loci and phenotypic variance and the usefulness of association mapping strategies depend on the extent of LD along the genome [66, 67] The extent of LD as a function of genetic distance is indicative of the depth of resolution as well as of the density of markers needed to obtain reliable results in association mapping studies [68].

Although either the absence of genetic distance information among markers or the fact that markers were widely distributed made it not possible to calculate the LD decay in our study, it was still possible to determine the level of genome-wide LD using a combination of AFLP and SNP markers. Based on non-syntenic SSR loci, [69] concluded that a 27.8% of the pairwise LD values was significant (p<0.01) in a durum wheat collection. This value was higher than the one obtained in our study (4.9%) using the highest number of markers available (134) in a combined SNP/AFLP analysis. Considering the entire collection, the number of available SNP was low (26 SNP) and additional analyses should be conducted to be conclusive. Furthermore, [70] obtained 14.4% of marker pairs in significant LD and a total average LD value between pairwise of non-syntenic loci of *r*^*2*^ = 0.029 using 592 DArT markers in a durum wheat panel. This value was higher than our estimation obtained with the combined analysis (AFLP and SNP) in the 119 accessions (*r*^*2*^ = 0.016). In conclusion, the low average LD value observed is an indication of the suitability of our collection to carry out association mapping studies. According to [25], a germplasm collection with low genomic LD is an important starting point for association mapping.

### What books tell us about durum wheat breeding and what DNA markers show us

#### Durum wheat breeding in Argentina

The first durum wheat seeds–mostly landraces with a low degree of variability–arrived in Argentina simultaneously with the arrival of immigrants [71]. The first breeding efforts made in the south of Buenos Aires province were centered on plant selections from these foreign populations, the first of which came from the Crimean peninsula. In particular, the durum wheat populations collected from the Russian port of Taganrog were characterized by the presence of tall plants with black awns, a spring growing habit and late heading time. Duro Capa, the first cultivar obtained in Argentina in 1926 by breeders of the Criadero Klein Company, was a cultivar with poor diffusion until 1931. Other companies, such as Buck Semillas, Vilela Fideos and La Previsión Experimental Station obtained their first cultivars through plant selections from the populations originally imported to Argentina. Between 1920 and 1930, the second Argentinian durum wheat breeding program was implemented by the Cooperativa de Seguros La Previsión located in Tres Arroyos, Buenos Aires province (now INTA CEI Barrow). After a few years of selections and field evaluations the first cultivar–named Candeal Selección La Previsión–was released in 1939. In the next two decades, a new germplasm was introduced from Russia, USA and Europe, particularly from Italy, and the first crosses were performed. In 1952, Buck Semillas released Candeal Durumbuck and during 1961 and 1966 two new selections from Taganrog (Taganrog Sel. Buck and Taganrog Vilela Fideos) and the first cultivar from the CEI Barrow breeding program, Candeal Bonaerense 202, were released, respectively.

With the advent of the green revolution, the germplasm from the International Maize and Wheat Improvement Center (CIMMYT) was widely disseminated around the world. Semi-dwarf plants with better performance than landraces or tall cultivars, rapidly gained position into the breeding programs. The highest adoption rate in Latin America was during the period 1966–1990 [72]. CIMMYT’s durum wheat began to be tested in Argentina during the ´70s and Balcarceño INTA was one of the first durum wheat genotypes which incorporated semi-dwarf genes from CIMMYT sources. The adoption of semi-dwarf varieties in Argentina ranged from 18% (1977) to 100% (1989) [72]. Also, during the ´70s, new Italian genetic resources (Gerardo group) were received at INTA CEI Barrow and in 1979/1980 the selection Gerardo 516 was released as Bonaerenese Valverde. On the other hand, the cultivar Taganrog Buck Balcarce (1980) incorporated Senatore Cappelli into Argentinian durum wheat pedigree. Later, the cultivar Buck Topacio (1997) introduced from University of Hohenheim was cultivated during at least 20 years. From the ´80s until now, most of the breeding process has been dominated by the release or use of germplasm improved by CIMMYT and some varieties received mainly from France, Germany and Italy to increase genetic variability through new crosses.

#### Population structure and clustering analyses among accessions

Genetic relationships in our durum wheat collection were analyzed by means of different statistical methods to assess genetic diversity level and population structure. The genetic contribution of foreign germplasms to the Argentinian breeding programs was also explored, yielding a valuable insight into germplasm introduction along the breeding process. Clustering results obtained when using molecular markers can be affected depending on the number and type of markers, sample size and the cluster algorithm applied [73]. In our study, both AFLP and SNP markers provided useful and complementary information about the genetic relationships in the collection studied. Although the AFLP markers are inherited in a dominant Mendelian fashion, they were observed to have a better ability than the SNP markers to discriminate sister lines. The possibility of a bias effect as a result of the number of AFLP markers used should, nonetheless, not be discarded. The differences observed between AFLP and SNP markers to determine population structure probably result from mutational properties of DNA which are differently captured by these two marker types. Increasing the number of SNP markers will allow us to perform a deeper genotyping of our durum wheat collection and will guarantee a better discrimination of highly related genotypes.

The analysis using SNPs allowed us to detect two main subpopulations (*K* = 2) in the entire collection. The results derived from PCoA, the clustering distance-based method (UPGMA) and the Bayesian clustering approach performed using the STRUCTURE software were congruent to assign genotypes (87.5%) to one of these two main subpopulations. A general evaluation of these two subpopulations divided the entire collection into two main germplasm sources. The subpopulation 1 (SbpS_1), which included germplasm with highest CIMMYT influence, corresponded to: i) crosses recorded in different countries (Argentina, Chile, Cyprus) but developed in Mexico, ii) genotypes with CIMMYT parents in their pedigree, iii) genotypes related with the CIMMYT breeding program through the ICARDA international center, such as those from the WANA region, and iv) CIMMYT nursery material included in our collection. Between founder CIMMYT materials, the cultivar Altar 84 was the more frequently observed in the pedigree of the different accessions. Other CIMMYT genotypes, such as Yavaros 79, Mexicali 75 and Flamingo also formed part of the pedigrees of accessions with CIMMYT origin or ancestry. The supremacy of CIMMYT germplasm in Argentinian pedigrees is slowly decreasing as a result of the presence of new genetic sources from France, Germany, Italy and ICARDA. The economic impact of semi-dwarf cultivars was measured in terms of productivity by [74] whose results indicate that CIMMYT has contributed with approximately 53.77 kg/ha per year during 1962–2002. The adoption of CIMMYT related genotypes was highest in Latin America than in other regions. According to [75], 70% of the spring durum wheat varietal releases during 1994–2014 in Latin America included CIMMYT breeding lines used directly. Apart from the beneficial effects of *Rht* genes, CIMMYT germplasm was characterized by its wide adaptation, short life-cycle and high yield potential.

Our collection has a limited number (4) of durum wheat accessions from USA which were clustered in SbpS_1, including two Langdon-derived genotypes. However, four Kofa derivative genotypes and seven crosses directly involving founder genotypes from North-Dakota (USA), such as Cando and Lakota, were also included as part of this subpopulation.

The subpopulation 2 (SbpS_2) was composed of accessions from the European Mediterranean basin and Argentinian cultivars or breeding lines with influence from this region, mainly from Italian germplasm. The Taganrog derivative genotypes were also part of this subpopulation. Most of the traditional accessions, except for two from Argentina and Italy, were included in the SbpS_2 subpopulation. The two traditional accessions Buck Mechongue and Duilio clustered in the subpopulation 1, explained by the clear influence of CIMMYT on their pedigrees. The fact that traditional accessions from both countries were grouped together was also supported by our PCoA analysis performed taking into account the origin of genotypes and using both AFLP and SNP markers. Traditional and modern accessions from Italy and Argentina were deliberately separated in our analyses to test their relationships taking into account the historical records previously described. The Italian germplasm was widely spread all over the world in the first years of the twentieth century, specially the most successful cultivar ‘Senatore Cappelli’ which was released in 1915 [76]. The contribution of Cappelli to the Italian germplasm is well documented [77] and can be verified observing the pedigree of the genotypes used in our study. On the other hand, the effects of introducing the Gerardo group into national breeding programs, especially into INTA program, can be traced to the present time by analyzing the last released cultivar registered by INTA, BonINTA Quillen, which includes Bonaerense Valverde (GDO VZ516). The SbpS_2 subpopulation included other old materials from WANA region, such as Etit 38 and Haurani, as well as Haurani derivative cultivars from the Om Rabi group. According to [77], the Om Rabi group was one of the first crosses produced by ICARDA and it is still cultivated in some countries. Both Etit 38 and Haurani together with Taganrog are considered the only three landraces present in our collection. To our knowledge, Taganrog can nonetheless be considered a founder genotype of Argentinian germplasm and can be included within the group of traditional Argentinian accessions. Several founder cultivars from Italy, Middle East, and Nord America were described by [1,9], among them Haurani, Cappelli, Apullo, Creso, Altar 84, Langdon and Lakota, which were also included in our collection as part of the pedigrees or directly as accessions. Most of the French germplasm was also included in this subpopulation, mainly corresponding to modern materials.

The data derived from our structure analysis using either AFLP markers (*K* = 6) or SNP markers (*K* = 5) showed a fine tuning division among accessions. In spite of the different *K* values identified, this analysis allowed us to detect substructure layers with more closely related accessions. The number of accessions having a mixed genetic structure was higher using SNP (*K* = 5) than using AFLP markers (*K* = 6), probably due the AFLP number or their dominant fashion.

In view of the above, it could be concluded that the genotypes from Cyprus and Chile are strongly associated with CIMMYT germplasm, being part of the genetically related clusters SbpS_2 and SbpS_3 in the *K* = 5 model using SNP. This was also evidenced by the PCoA analysis based on geographical origin. Modern Argentinian accessions were included in these two subpopulations although they were clustered in SbpS_1 and mainly in SbpS_4 associated with Mediterranean accessions, evidencing a higher variance of our germplasm.

On the other hand, according to the AFLP analysis the modern Argentinian genotypes were clustered between SbpA_1 and SbpA_4 subpopulations although the major part was included within SbpA_6. The founder effect of the Gerardo group on Argentinian genotypes could be observed in SbpS_1 and also in SbpA_4 subpopulations using SNP (SbpS) and AFLP makers (SbpA), respectively.

Based on the clusters with a higher number of modern Argentinian accessions (SbpS_4 and SbpA_6), the SNP markers were observed to have a better performance than the AFLP markers and they were also found to have the ability to clearly differentiate SbpS_4 (72.5% ARM) from other clusters. In addition, the position of SbpS_4 shown in the PCoA plot based on SNP markers suggests that part of the Argentinian germplasm took a different breeding direction. Most of the modern genotypes (10 out of 11) from Buck Semillas Company were included in this group. On the other hand, the AFLP markers maximized the differences among the subpopulations that contained French genotypes (SbpA_5) and among those from WANA region (SbpA_3). The genetic differences shown by Taganrog and their more direct derivative genotypes separated them in an independent subpopulation (SbpA_2), dividing the traditional Argentinian mainly into two clusters (SbpA_2 and SbpA_4).

Other authors [1] reported six main subpopulations when analyzing 134 durum wheat accessions and found a genetic differentiation between the Mediterranean germplasm from the CIMMYT-ICARDA accessions. A genetic divergence between Italian and CIMMYT/ICARDA germplasm was also clearly established by [77]. Further research identified founder genotypes in two durum wheat panels [9, 78]. In contrast, the structure analysis conducted by [70] in a tetraploid wheat collection mainly separated different tetraploid sub-species from the cultivated durum wheat accessions (*Triticum turgidum* var *durum*).

Our results supported by the AMOVA analyses revealed that most of the variance observed was due to differences among the genotypes within clusters, both within origins and genetic subpopulations. Similar findings were reported by other authors [8, 29, 77]. Therefore, compared to the initial origin-based analysis, our AMOVA based on genetic subpopulations maximized the differences among groups.

### Genetic diversity

Diversity index estimates in the entire collection were calculated using SNP markers. In Addition, a genetic diversity analysis was conducted in a subset of 119 accessions to compare the ability of AFLP and SNP markers to capture genetic variance. The mean of the expected heterozygosity calculated by AFLPs, *He* = 0.352 [35], also called PIC, was–on the one hand–similar to the average value reported by [65] and [5] but higher than that obtained by [79]. The mean *He* value calculated in the entire collection and the subset using SNPs were similar (0.183 and 0.182). The mean value of effective number of alleles (Ne) was higher for AFLP than for SNP markers, thus indicating that AFLP alleles were distributed more evenly across the subset than SNP markers, which also agrees with the lower number of rare alleles. All the variability indices obtained using AFLP markers were high, while the biased effect as a result of the number of markers used should not be discarded. The differences observed in the index values analyzed using AFLP and SNP markers decreased when indices were calculated considering only the filtered markers. The mean *He* values for 108 AFLP and 26 SNP markers were 0.377 and 0.348, respectively. This demonstrates that the MAF filtering had a higher effect on SNP markers than on AFLP markers not only in the number of markers retained but also in the diversity indices values. It could therefore be hypothesized that the two markers used to calculate genetic distances and to run the Bayesian clustering approach differed in number but not in the amount of variability captured per marker. The recommendations of [54] could therefore be considered correct for AFLP but not for set of SNP markers, particularly when the latter are used in a low number. Varshney et al. [17] reported a mean PIC value of 0.341 for 18 SNP markers in barley, which is quite similar to that obtained in our study using 26 SNP markers. From the point of view of the Argentinian germplasm, the genetic diversity observed in our collection is useful to be incorporated into national breeding programs.

On the other hand, the mean *Gst* values were moderate and low for AFLP (0.225) and SNP markers (0.131), respectively. Other authors reported a *Gst* = 0.173 using 44 SSRs in 172 landraces [8], i.e. an intermediate value among those obtained in the present study. The low level of genetic differentiation among subpopulations indicated by *Gst* values also agrees with the AMOVA results obtained using AFLP and SNP markers. Most of the genetic variability observed in our study was within subpopulations, with values of 81% in the subset of accessions using AFLP markers and of 67% in the entire collection calculated with SNP. Similar results were reported by [77] using 500 filtered SNP markers (68.3% within populations). This indicates that the number of SNP markers used in our work was suitable to estimate genetic variance. Maccaferri et al. [29] recorded 79.5% of variance within durum wheat subpopulations using SSRs whereas 81% of the variance detected in a worldwide bread wheat collection was among accessions within subpopulations [80]. The *Gst* parameter calculated per locus was not always correlated with the level of *He*, indicating that when a high number of markers is used, as for example that used in array technologies, *Gst* could be considered as a filtering criterion to maximize subpopulation differentiation capacity instead of the *He* value.

## Remarks and conclusions

AFLP and SNP markers were successfully applied to characterize a new durum wheat collection. This comprehensive study has also allowed us to establish not only the germplasm structure but also the major genetic relationships among accessions and to reconstruct a large part of the history of the durum wheat breeding process in Argentina during the last 80 years. More recently, international cooperation initiatives, such as the ´Wheat Initiative´ and its derivative projects accelerated and increased germplasm exchanges at a global scale. New and diverse sources of variability are currently being incorporated in the National durum wheat breeding programs.

## Supporting information

S1 Fig Evanno test used to estimate the true *K* obtained from STRUCTURE software. (A) Results obtained using 168 accessions analyzed with 26 SNP, (B) using 119 accessions analyzed with 26 SNP and, (C) using 119 accessions analyzed with 108 AFLP.
(PDF)

S2 Fig Comparison of phylogenetic trees obtained by UPGMA and Neighbor-Joining cluster methods in the whole durum wheat collection. (TIF)

S3 Fig Principal Coordinate Analysis (PCoA) based on genetic distance values calculated with 26 SNP markers in the entire durum wheat collection. PCoA among accessions based on binary genetic distance. (A) Subpopulations (SbpS) indicated by colors according the STRUCTURE results for *K* = 2, (B) and *K* = 5. (C) PCoA among subpopulations for *K* = 5 based on Nei's genetic distance values. A mixed subpopulation is that which is composed of accessions with an MP lower than 0.5 in all subpopulations. (D) PCoA calculated from Nei's genetic distance among the different geographical origins. Accessions are coded as ARM, modern Argentinian; ART, traditional Argentinian; CHI, Chile; CIM, CIMMYT; CYP, Cyprus; FRA, France; ITM, modern Italian; ITT, traditional Italian; USA, United States; WAN, West Asia North Africa region. Accessions from Argentina and Italy were divided into two groups according to the year of release (until and after 1985). Accessions labeled as "traditional" are those either bred or released until 1985.
(PDF)

S4 Fig Principal Coordinate Analysis (PCoA) performed using AFLP markers based Nei's genetic distance in a subset of 119 accessions. (A) PCoA among subpopulations according to STRUCTURE software (*K* = 6). (B) PCoA among the different geographical origins in the subset. Accessions are coded as ARM, modern Argentinian; ART, traditional Argentinian; CIM, CIMMYT; FRA, France; ITM, modern Italian; ITT, traditional Italian; USA, United States; WAN, West Asia North Africa region. Accessions from Argentina and Italy were divided into two groups according to the year of release (until and after 1985). Accessions labeled as "traditional" are those either bred or released until 1985.
(TIF)

S5 Fig Pie graph of percentage of molecular variance. Percentage of molecular variance explained by 108 AFLP markers in the subset of 119 accessions, within and among geographical origins of the accessions (A) and within and among subpopulations for *K* = 6 (B). Percentage of molecular variance explained by 26 SNP in the entire durum wheat collection considering the geographical origin of accessions (C) and within and among subpopulations for *K* = 5 (D).
(TIF)

S1 Table List of KASP markers used in this study and results. (XLSX)

S2 Table Accessions assigned per subpopulation by STRUCTURE software using 26 SNP markers in the entire durum wheat collection. (A) Analysis for *K* = 2 model. (B) Analysis for *K* = 5 model.
(XLSX)

S3 Table Accessions assigned to each subpopulation by STRUCTURE software for *K* = 6 model (maximum *ΔK*) using 108 AFLP markers in the subset of 119 accessions. (XLSX)

S4 Table Percentage of variation explained by the first 3 axes of PCoA and pairwise Nei's genetic distance and identity calculated using 26 SNP markers in the entire durum wheat collection. (A) PCoA among accessions calculated with 26 SNP in the whole durum wheat collection, (B) Pairwise Nei's genetic distance and identity among subpopulations (C) PCoA among subpopulations, (D) Pairwise Nei’s genetic distance and identity values among the geographical origins of accessions, (E) PCoA among the geographical origins of accessions in the entire durum wheat collection.
(XLSX)

S5 Table Accessions assigned to each subpopulation by different methodologies at *K* = 2 using 26 SNP markers in a durum wheat collection. (XLSX)

S6 Table Pairwise Nei's genetic distance and identity among the origins of accessions and subpopulations calculated using 108 AFLP markers in the subset of 119 accessions and percentage of variation explained by the first 3 axes of PCoA. (A) Pairwise Nei's genetic distance and identity among subpopulations, (B) PCoA among subpopulations in the subset of 119 accessions, (C) Pairwise Nei's genetic distance and identity among the origins of accessions, (D) PCoA among the origins of accessions in the subset of 119 accessions.
(XLSX)

S7 Table Pairwise *Fst* values obtained using 26 SNP markers and genetic diversity indices estimated using 56 SNP markers in the entire durum wheat collection. (A) Pairwise *Fst* values among subpopulations, (B) Genetic diversity among origins, (C) Pairwise *Fst* values among the geographical origins of accessions.
(XLSX)

S8 Table Allele frequencies and genetic diversity indices calculated using AFLP and SNP markers and considering the subpopulations or the geographical origins of accessions in the subset of 119 accessions. (A) per locus using 125 AFLPs, (B) per subpopulation using 125 AFLPs, (C) by origin using 125 AFLP, (D) per locus using 56 SNPs, (E) per subpopulation estimated using 56 SNPs.
(XLSX)
