# Supplementary figures and images for: Genetic diversity and linkage disequilibrium using SNP (KASP) and AFLP markers in a worldwide durum wheat (Triticum turgidum L. var durum) collection
Source: PLoS One. 2019 Jun 28;14(6):e0218562. doi: 10.1371/journal.pone.0218562 (PMC6741835; doi:10.1371/journal.pone.0218562)

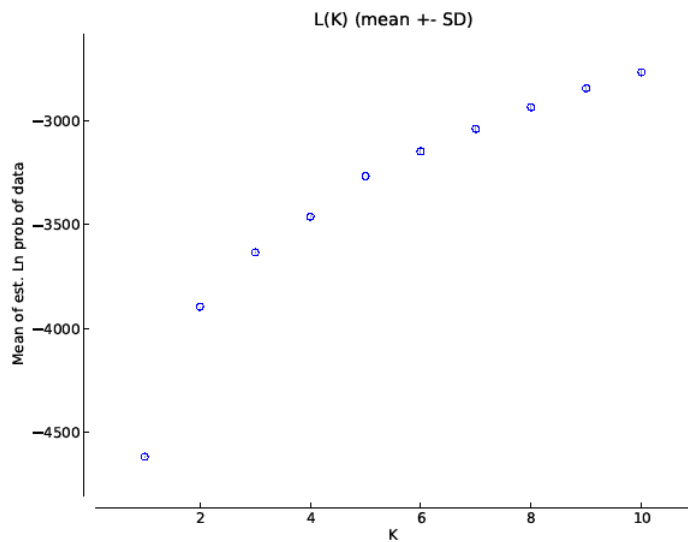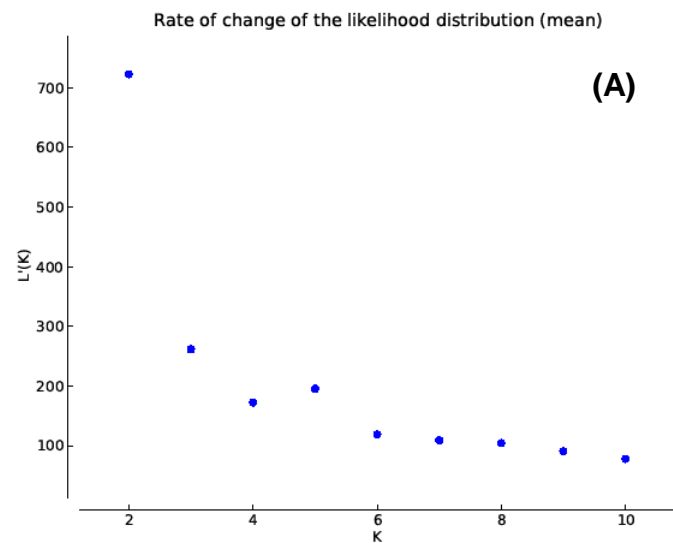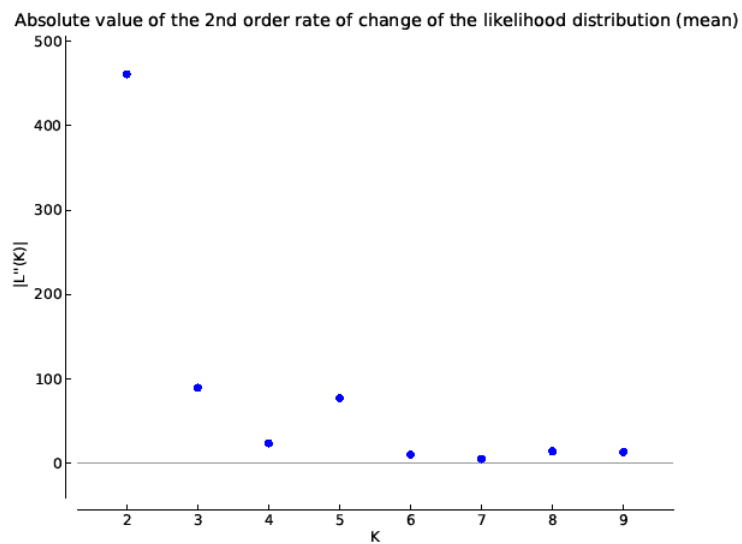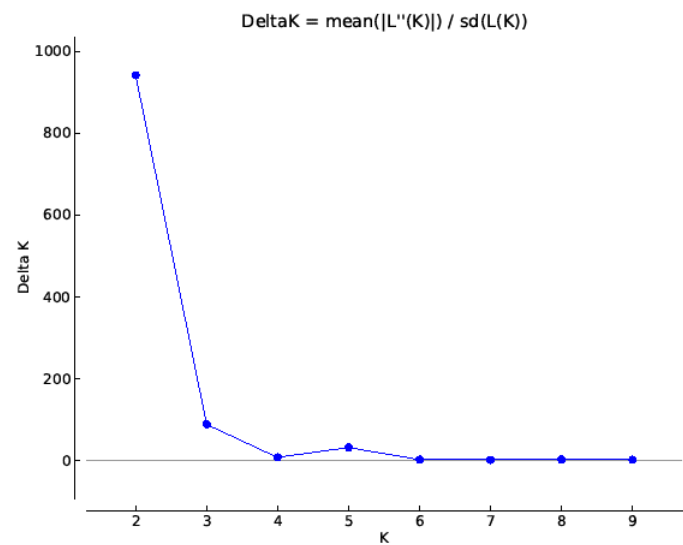

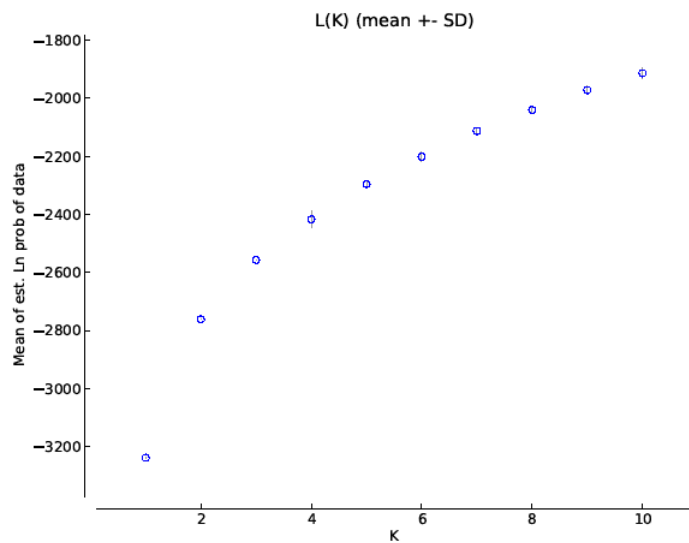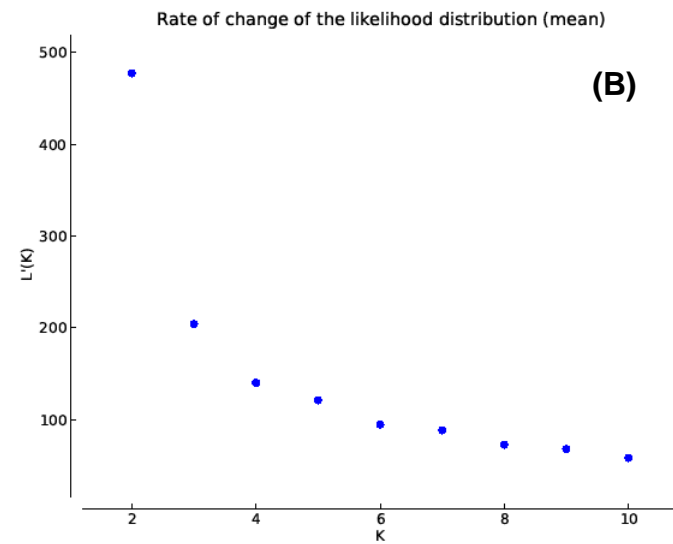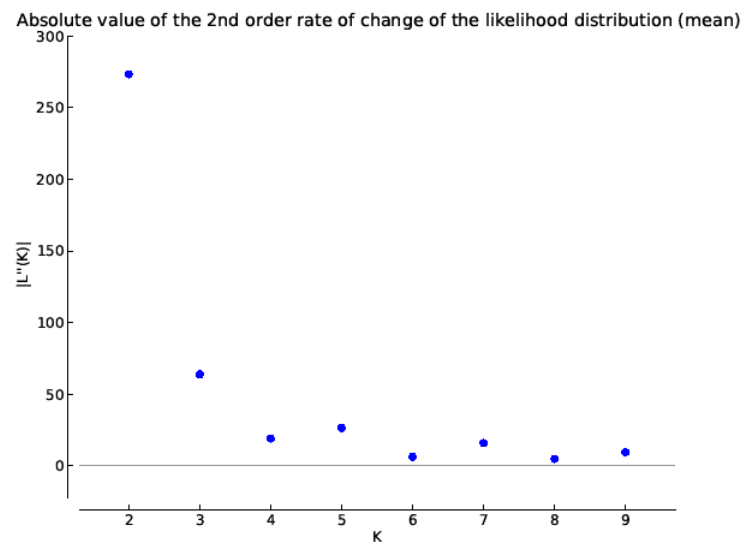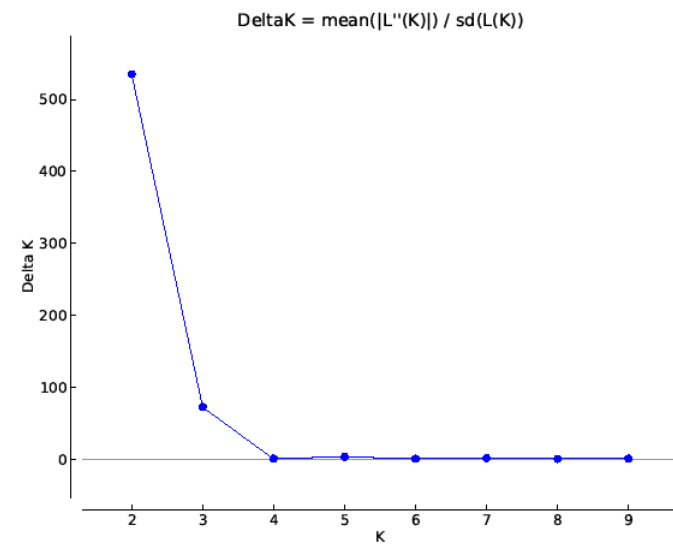

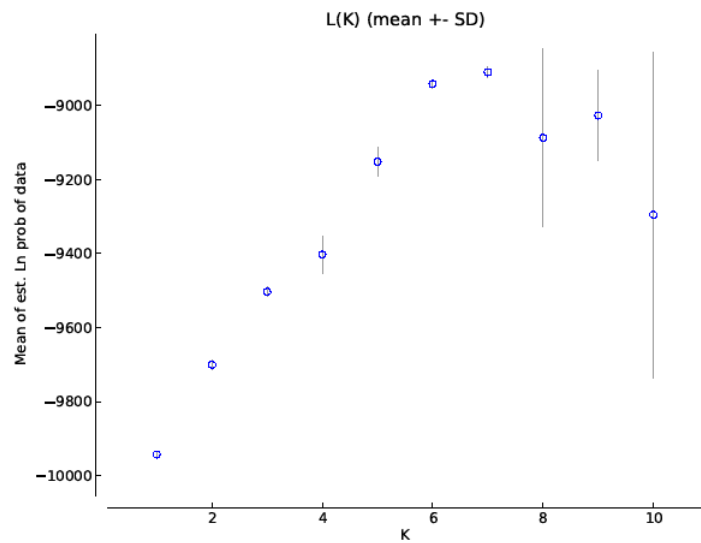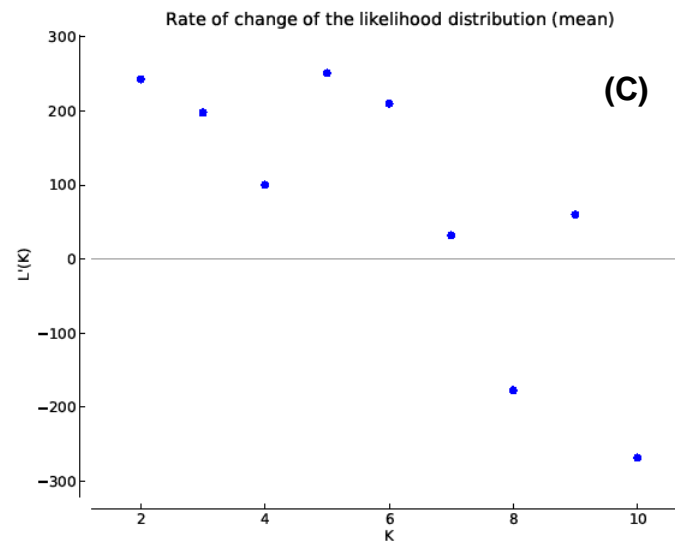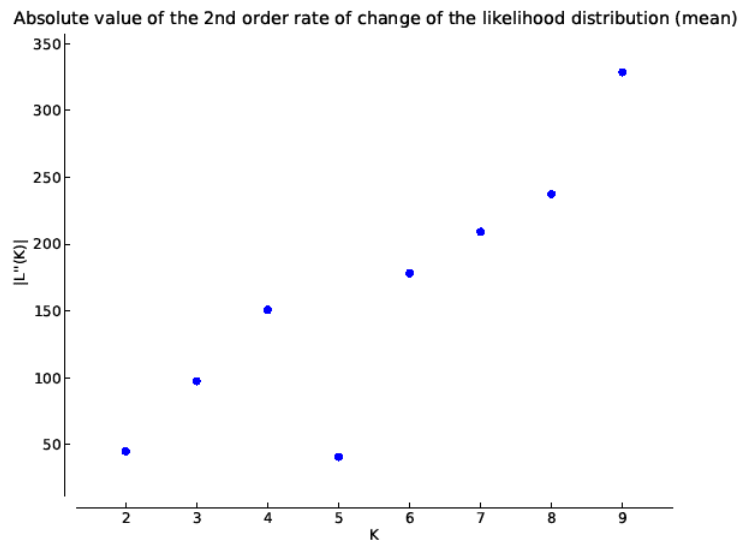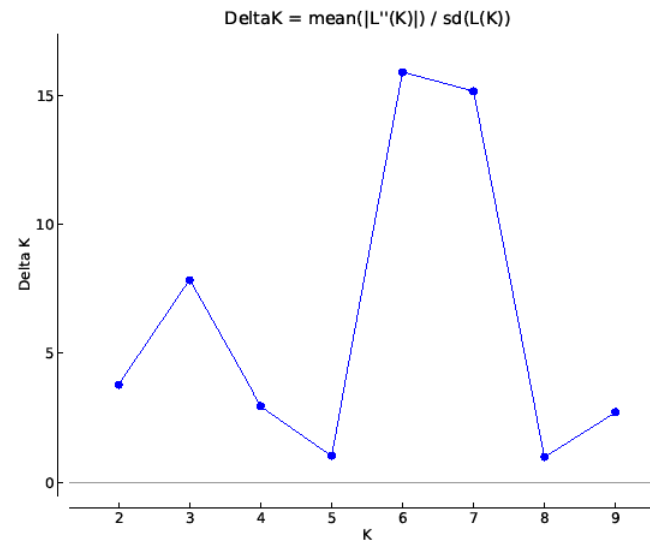

Supplement: S1 Fig — (A) Results obtained using 168 accessions analyzed with 26 SNP, (B) using 119 accessions analyzed with 26 SNP and, (C) using 119 accessions analyzed with 108 AFLP. (PDF) [file pone.0218562.s001.pdf]

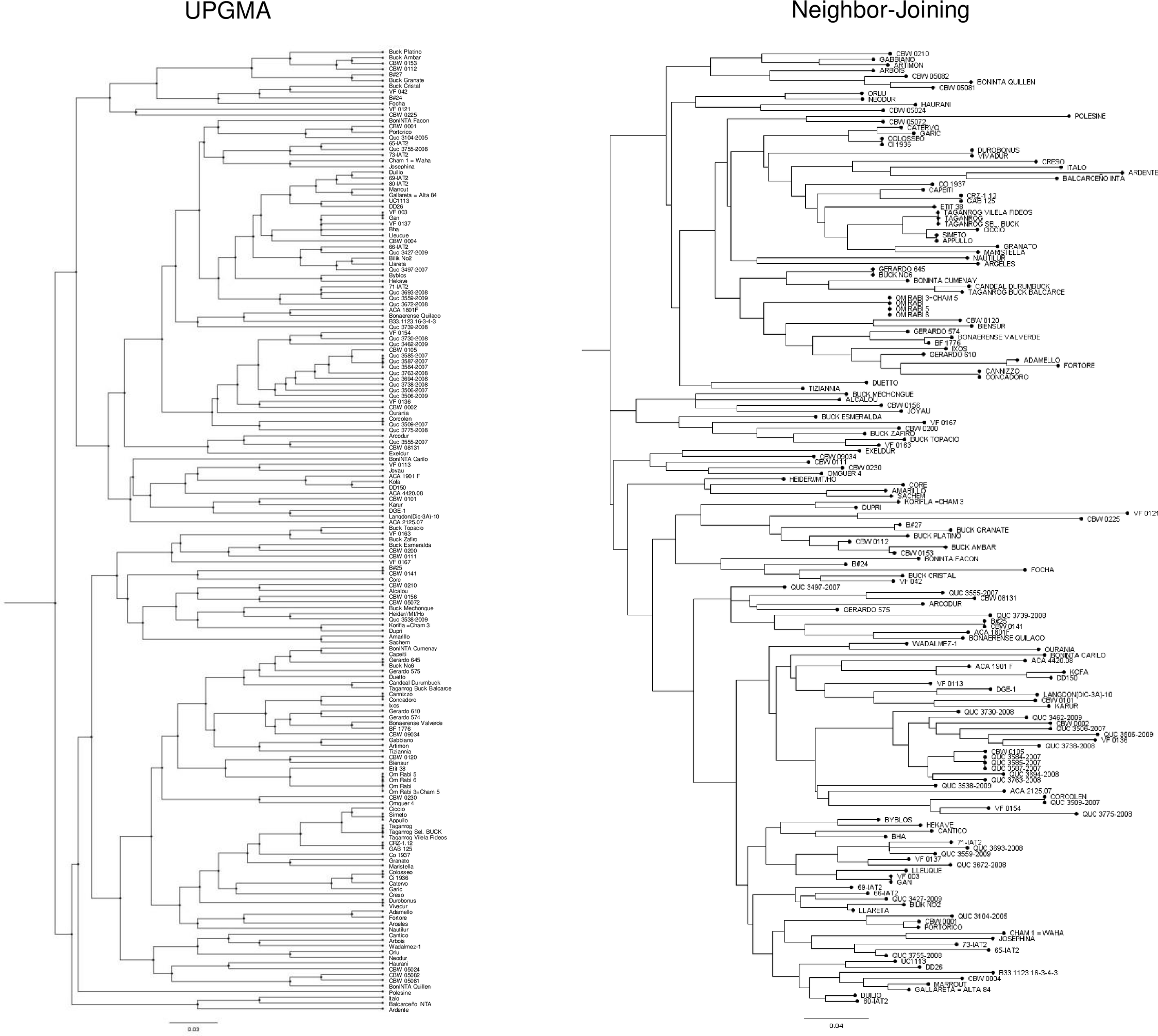

Supplement: S2 Fig — (TIF) [file pone.0218562.s002.tif]

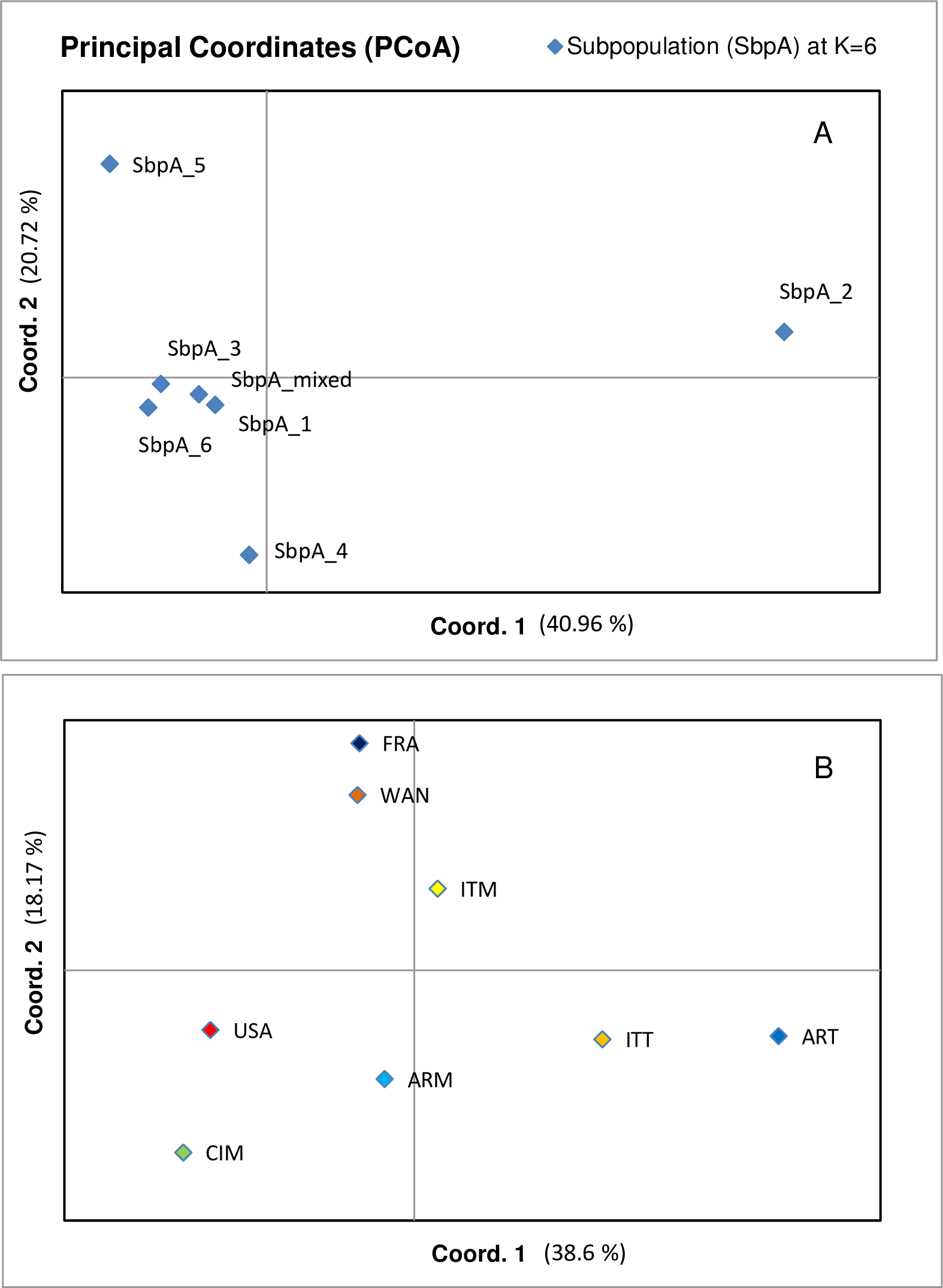

Supplement: S4 Fig — (A) PCoA among subpopulations according to STRUCTURE software (K = 6). (B) PCoA among the different geographical origins in the subset. Accessions are coded as ARM, modern Argentinian; ART, traditional Argentinian; CIM, CIMMYT; FRA, France; ITM, modern Italian; ITT, traditional Italian; USA, United States; WAN, West Asia North Africa region. Accessions from Argentina and Italy were divided into two groups according to the year of release (until and after 1985). Accessions labeled as "traditional" are those either bred or released until 1985. (TIF) [file pone.0218562.s004.tif]

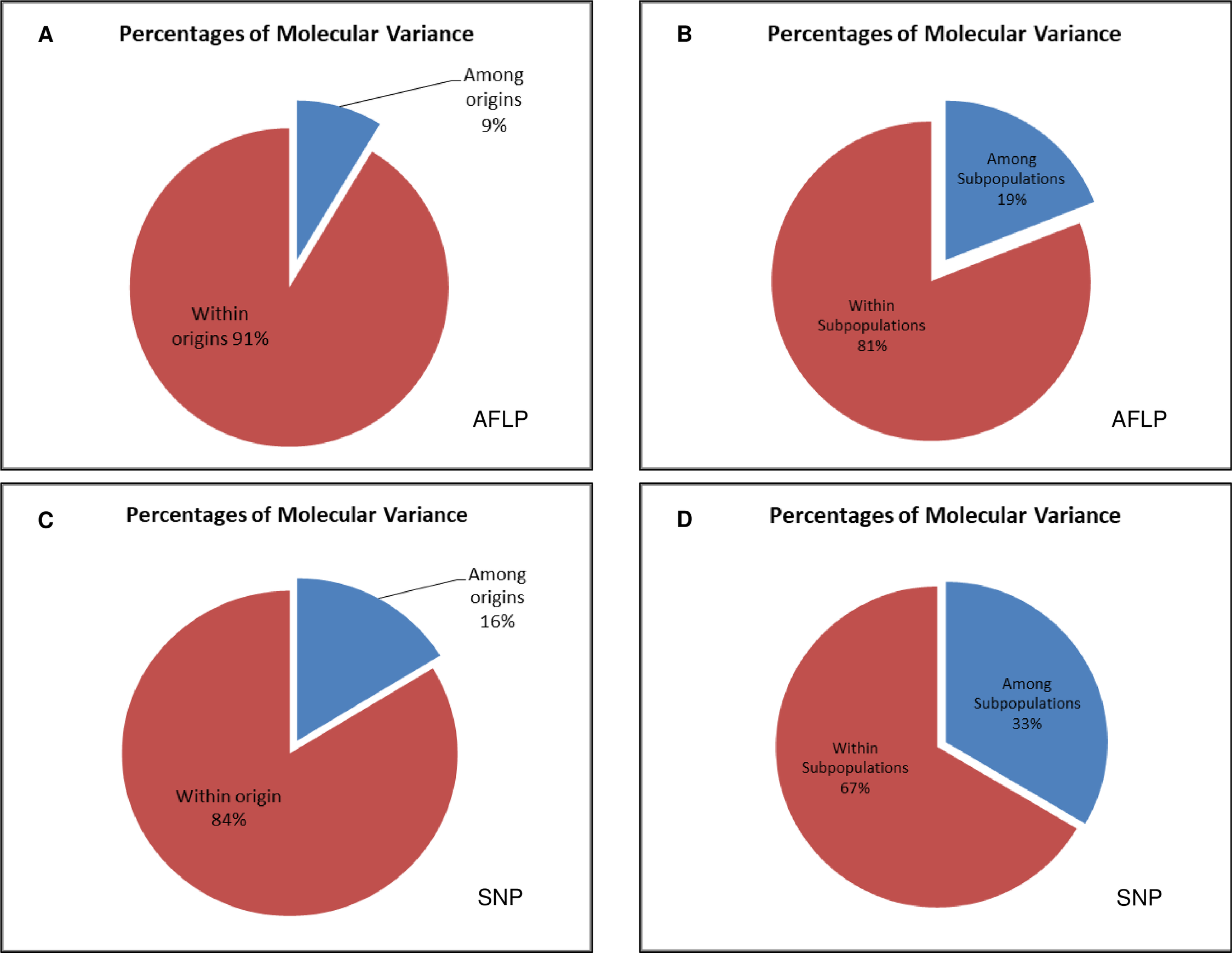

Supplement: S5 Fig — Percentage of molecular variance explained by 108 AFLP markers in the subset of 119 accessions, within and among geographical origins of the accessions (A) and within and among subpopulations for K = 6 (B). Percentage of molecular variance explained by 26 SNP in the entire durum wheat collection considering the geographical origin of accessions (C) and within and among subpopulations for K = 5 (D). (TIF) [file pone.0218562.s005.tif]
