# Supplementary material for: Genetic diversity and linkage disequilibrium using SNP (KASP) and AFLP markers in a worldwide durum wheat (Triticum turgidum L. var durum) collection
Source: PLoS One. 2019 Jun 28;14(6):e0218562. doi: 10.1371/journal.pone.0218562 (PMC6741835; doi:10.1371/journal.pone.0218562)
Supplement: S3 Fig — PCoA among accessions based on binary genetic distance. (A) Subpopulations (SbpS) indicated by colors according the STRUCTURE results for K = 2, (B) and K = 5. (C) PCoA among subpopulations for K = 5 based on Nei's genetic distance values. A mixed subpopulation is that which is composed of accessions with an MP lower than 0.5 in all subpopulations. (D) PCoA calculated from Nei's genetic distance among the different geographical origins. Accessions are coded as ARM, modern Argentinian; ART, traditional Argentinian; CHI, Chile; CIM, CIMMYT; CYP, Cyprus; FRA, France; ITM, modern Italian; ITT, traditional Italian; USA, United States; WAN, West Asia North Africa region. Accessions from Argentina and Italy were divided into two groups according to the year of release (until and after 1985). Accessions labeled as "traditional" are those either bred or released until 1985. (PDF) [file pone.0218562.s003.pdf]

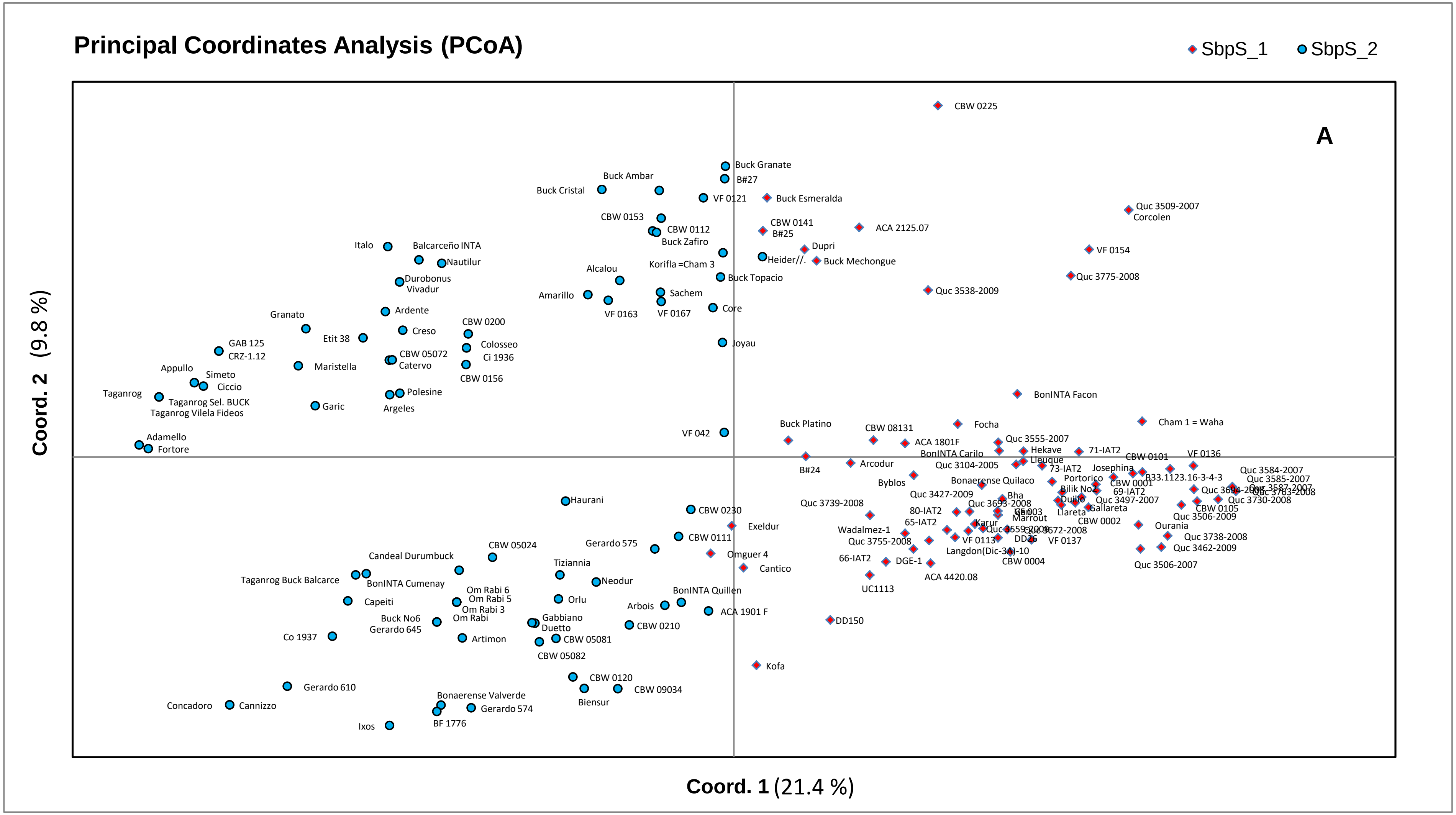
Principal Coordinates Analysis (PCoA)
SbpS_1
SbpS_2
A
Coord. 2 (9.8 %)
Coord. 1 (21.4 %)
CBW 0225
Buck Granate
B#27
Buck Ambar
Buck Cristal
VF 0121
Buck Esmeralda
Quc 3509-2007
Corcolen
CBW 0153
CBW 0141
B#25
ACA 2125.07
CBW 0112
Buck Zafiro
Italo
Balcarceño INTA
Nautilur
Dupri
VF 0154
Korifla =Cham 3
Heider//.
Buck Mechongue
Alcalou
Durobonus
Vivadur
Buck Topacio
Quc 3775-2008
Amarillo
Sachem
Quc 3538-2009
Granato
Ardente
VF 0163
VF 0167
Core
CBW 0200
Creso
Etit 38
GAB 125
CRZ-1.12
Colosseo
Ci 1936
Joyau
CBW 05072
Catervo
Maristella
Appullo
Simeto
Ciccio
CBW 0156
Taganrog
Polesine
Taganrog Sel. BUCK
Taganrog Vilela Fideos
Garic
Argeles
BonINTA Facon
Buck Platino
Focha
Cham 1 = Waha
CBW 08131
VF 042
Adamello
Fortore
ACA 1801F
Quc 3555-2007
BonINTA Carilo
Hekave
71-IAT2
Lleuque
CBW 0101
VF 0136
B#24
Arcodur
Quc 3104-2005
73-IAT2
Josephina
Quc 3584-2007
Byblos
Bonaerense Quilaco
Portorico
B33.1123.16-3-4-3
Quc 3585-2007
CBW 0001
Bilik No2
69-IAT2
Quc 3694-2009
Quc 3587-2007
Quc 3763-2008
Quc 3427-2009
Bha
Duilio
Quc 3497-2007
Quc 3730-2008
Quc 3739-2008
Quc 3693-2008
Gallareta
CBW 0105
Haurani
CBW 0230
80-IAT2
CF003
Llareta
Quc 3506-2009
Marrout
CBW 0002
65-IAT2
Karur
Ourania
Exeldur
Wadalmez-1
Quc 3559-2009
Quc 3672-2008
CBW 0111
Quc 3755-2008
VF 0113
DD76
VF 0137
Quc 3738-2008
Gerardo 575
Langdon(Dic-34)-10
Quc 3462-2009
CBW 05024
Omguer 4
66-IAT2
DGE-1
CBW 0004
Candeal Durumbuck
Tiziannia
Cantico
Quc 3506-2007
ACA 4420.08
Taganrog Buck Balcarce
Neodur
BonINTA Cumenay
UC1113
Om Rabi 6
BonINTA Quillen
Om Rabi 5
Capeiti
Orlu
Om Rabi 3
Arbois
ACA 1901 F
Om Rabi
Buck No6
Gabbiano
Gerardo 645
Duetto
DD150
CBW 0210
Co 1937
Artimon
CBW 05081
CBW 05082
Kofa
CBW 0120
Gerardo 610
Bonaerense Valverde
CBW 09034
Concadoro
Cannizzo
Biensur
Gerardo 574
Ixos
BF 1776

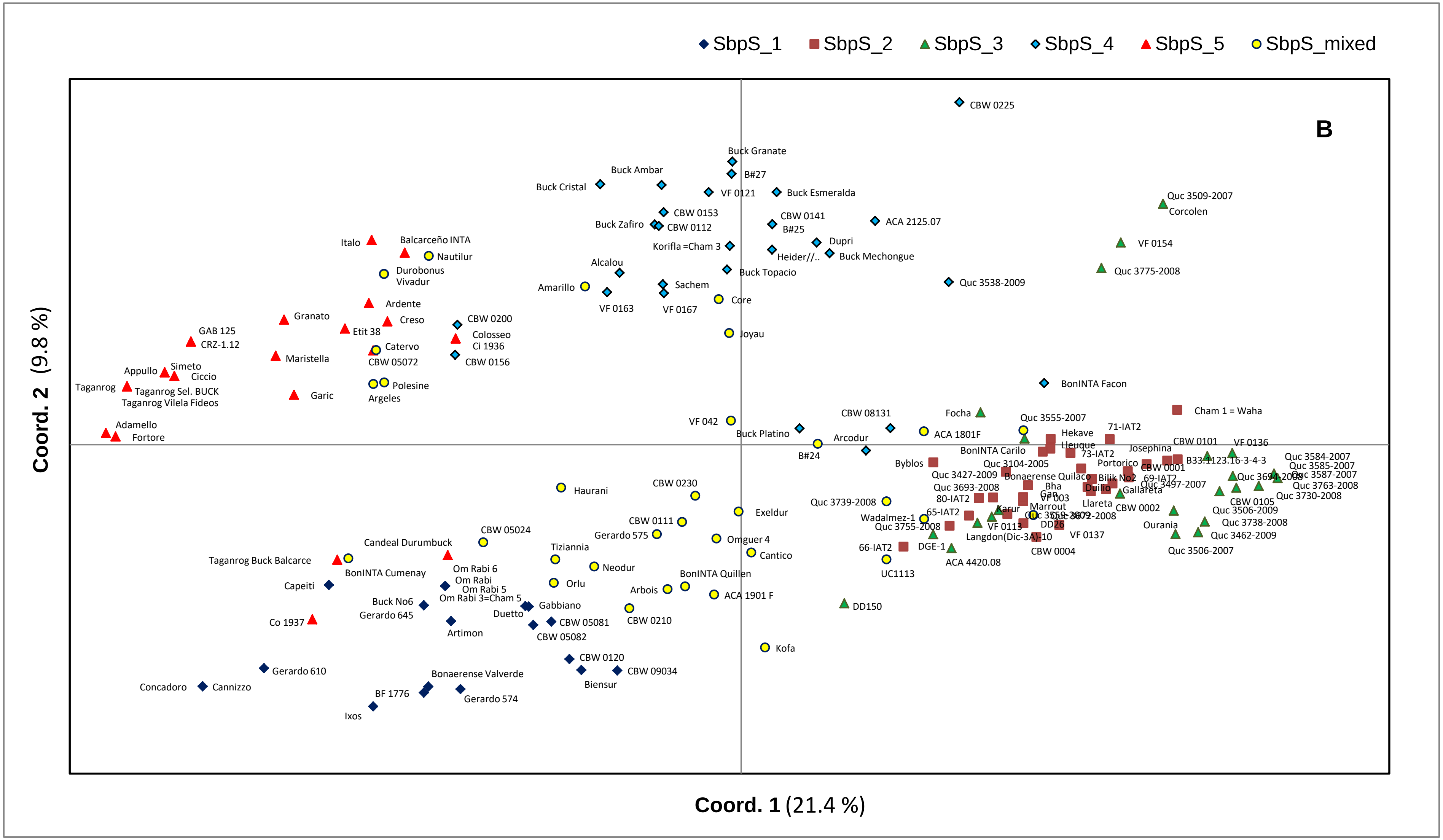
SbpS_1
SbpS_2
SbpS_3
SbpS_4
SbpS_5
SbpS_mixed
B
Coord. 2 (9.8 %)
Coord. 1 (21.4 %)
CBW 0225
Buck Granate
B#27
Buck Ambar
Buck Cristal
VF 0121
Buck Esmeralda
CBW 0153
CBW 0141
Buck Zafiro
CBW 0112
B#25
ACA 2125.07
Quc 3509-2007
Corcolen
Korifla =Cham 3
Dupri
Heider//..
Buck Mechongue
VF 0154
Italo
Balcarceño INTA
Nautilur
Alcalou
Buck Topacio
Quc 3775-2008
Durobonus
Vivadur
Quc 3538-2009
Amarillo
Sachem
VF 0163
VF 0167
Core
Ardente
Granato
Creso
CBW 0200
GAB 125
Etit 38
Colosseo
CRZ-1.12
Caterv
Ci 1936
Joyau
Maristella
CBW 05072
CBW 0156
Appullo
Simeto
Ciccio
Taganrog
Taganrog Sel. BUCK
Garic
Polesine
Argeles
Taganrog Vilela Fideos
BonINTA Facon
Adamello
Fortore
Focha
Cham 1 = Waha
CBW 08131
Quc 3555-2007
VF 042
Buck Platino
ACA 1801F
Hekave
71-IAT2
Arcodur
Lleuque
B#24
BonINTA Carilo
73-IAT2
Josephina
CBW 0101
VF 0136
Byblos
Quc 3104-2005
Portorico
B33-1123.16-3-4-3
Quc 3584-2007
Quc 3585-2007
CBW 0001
Quc 3427-2009
Bonaerense Quilaco
Bilik No2
69-IAT2
Quc 3694-2008
Quc 3587-2007
Haurani
CBW 0230
Quc 3693-2008
Bha
Duilio
Quc 3497-2007
Quc 3763-2008
Gallareta
80-IAT2
VF 003
Quc 3730-2008
Quc 3739-2008
Llareta
CBW 0105
Karur
Marrout
CBW 0002
Exeldur
65-IAT2
Quc 3506-2009
CBW 0111
Wadalmez-1
Quc 3738-2008
Quc 3755-2008
VF 0113
DD26
Ourania
CBW 05024
Gerardo 575
Omguer 4
Langdon(Dic-3A)-10
VF 0137
Quc 3462-2009
Candeal Durumbuck
Tiziannia
66-IAT2
DGE-1
Cantico
CBW 0004
Quc 3506-2007
Taganrog Buck Balcarce
Neodur
ACA 4420.08
Om Rabi 6
BonINTA Cumenay
Om Rabi
UC1113
BonINTA Quillen
Capeiti
Orlu
Om Rabi 5
Arbois
Om Rabi 3=Cham 5
ACA 1901 F
Buck No6
Gabbiano
DD150
Gerardo 645
Duetto
CBW 0210
Co 1937
CBW 05081
Artimon
CBW 05082
Kofa
CBW 0120
Gerardo 610
CBW 09034
Bonaerense Valverde
Biensur
Concadoro
Cannizzo
BF 1776
Gerardo 574
Ixos

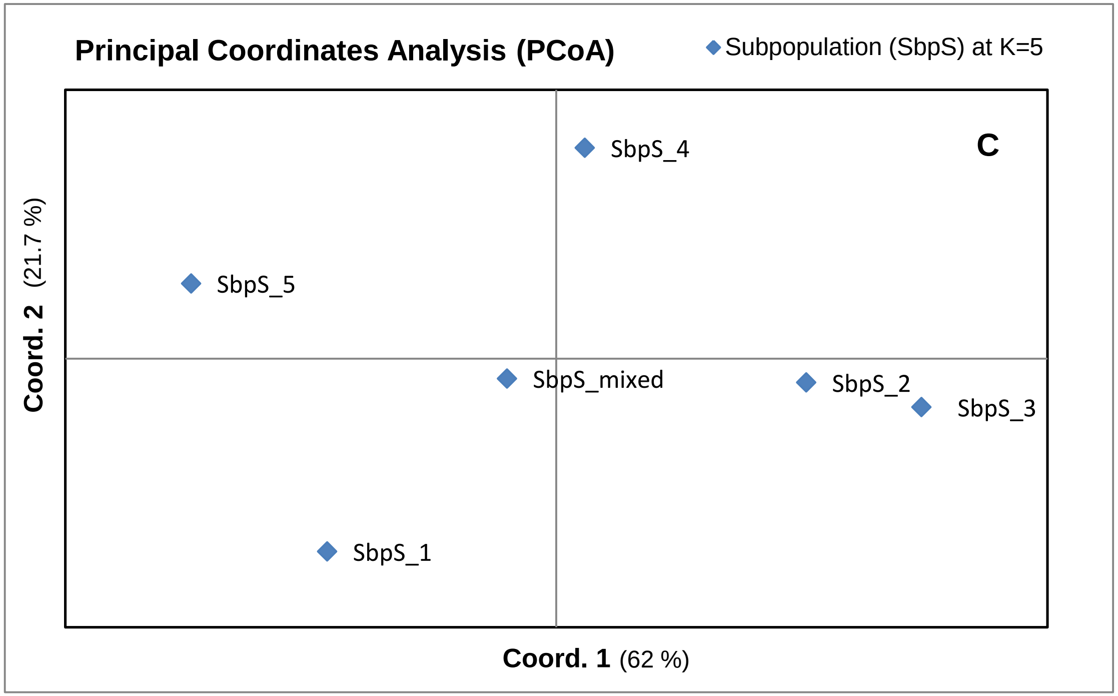

Principal Coordinates Analysis (PCoA)
Subpopulation (SbpS) at K=5
C
SbpS_4
SbpS_5
SbpS_mixed
SbpS_2
SbpS_3
SbpS_1
Coord. 2 (21.7 %)
Coord. 1 (62 %)

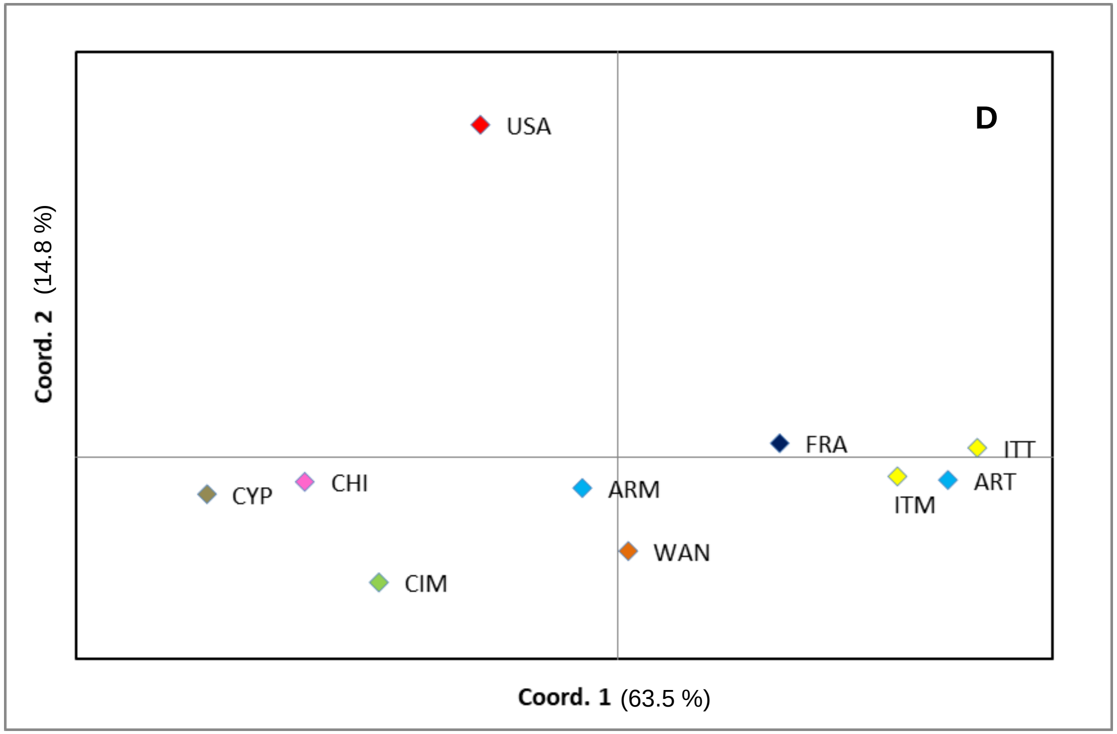

D
USA
FRA
ITT
CYP
CHI
ARM
ITM
ART
WAN
CIM
Coord. 2 (14.8 %)
Coord. 1 (63.5 %)
